# A Virtual Clinical Trial of Psychedelics to Treat Patients With Disorders of Consciousness

**DOI:** 10.1002/advs.202511780

**Published:** 2025-11-20

**Authors:** Naji L.N. Alnagger, Paolo Cardone, Charlotte Martial, Yonatan Sanz Perl, Iván Mindlin, Jacobo D Sitt, Leor Roseman, Robin Carhart‐Harris, David Nutt, Pablo Mallaroni, Natasha Mason, Johannes G Ramaekers, Vincent Bonhomme, Steven Laureys, Gustavo Deco, Olivia Gosseries, Pablo Núñez, Jitka Annen

**Affiliations:** ^1^ Coma Science Group, GIGA‐Consciousness University of Liège Avenue de l'hôpital 11 Liège 4000 Belgium; ^2^ NeuroRehab & Consciousness Clinic, Neurology Department University Hospital of Liège Avenue de l'hôpital 11 Liège 4000 Belgium; ^3^ Institut du Cerveau – Paris Brain Institute – ICM Inserm CNRS, Sorbonne Université Paris 75013 France; ^4^ Department of Information and Communication Technologies, Centre for Brain and Cognition, Computational Neuroscience Group Universitat Pompeu Fabra Barcelona 08005 Spain; ^5^ Department of Psychology University of Exeter Exeter EX4 4QG UK; ^6^ Centre for Psychedelic Research, Department of Brain Sciences, Faculty of Medicine Imperial College London London W12 0HS UK; ^7^ Psychedelics Division, Neuroscape University of California, San Francisco San Francisco CA CA 94158 USA; ^8^ Department of Neuropsychology and Psychopharmacology Faculty of Psychology and Neuroscience Maastricht University Maastricht 6229 ER The Netherlands; ^9^ Anesthesia and Perioperative Neuroscience Laboratory, GIGA‐Consciousness, GIGA Institute University of Liège Liège 4000 Belgium; ^10^ Department of Anaesthesia and Intensive Care Medicine Liège University Hospital Liège 4000 Belgium; ^11^ CERVO Brain Research Centre, Laval University 2601 de la Canardière Québec G1J 2G3 Canada; ^12^ Biomedical Engineering Group University of Valladolid Valladolid 47011 Spain; ^13^ Centro de Investigación Biomédica en Red en Bioingeniería, Biomateriales y Nanomedicina, (CIBER‐BBN) Madrid 28029 Spain; ^14^ Department of Data Analysis University of Ghent Henri Dunantlaan 1 Ghent 9000 Belgium

**Keywords:** criticality, diffusion‐weighted imaging (DWI), disorders of consciousness, functional magnetic resonance imaging (fMRI), psychedelics, whole‐brain modeling

## Abstract

Disorders of consciousness (DoC), including unresponsive wakefulness syndrome (UWS) and minimally conscious state (MCS), have limited treatment options and are characterized by low complexity of brain activity. Recent research suggests that psychedelic drugs, which enhance the complexity of brain activity, could offer promising therapies. Here, individualized whole‐brain computational models are developed for patients with DoC, optimized with empirical functional magnetic resonance imaging data and diffusion‐weighted imaging data, upon which the administration of lysergic acid diethylamide (LSD) and psilocybin is simulated. An in silico perturbation protocol is applied to assess brain dynamics, first distinguishing between different states of consciousness, including DoC, anesthesia, and the psychedelic state. Then, brain dynamics are assessed before and after a simulation of psychedelic drugs on patients with DoC. Findings indicated that the simulation of LSD and psilocybin shifted the brain activity of patients with DoC closer to criticality (the point at a phase transition between order and chaos), with a greater effect in patients in the MCS. In patients with UWS, the treatment response correlated with structural connectivity, while in patients in the MCS, it aligned with baseline functional connectivity. These results offer a computational foundation for using psychedelics in DoC treatment and highlight the potential future role of computational modeling in drug discovery and personalized medicine.

## Graphical abstract

Disorders of consciousness after severe brain injury are marked by reduced complexity of brain activity and limited treatment options. Using personalized whole‐brain models, this study shows that simulated lysergic acid diethylamide (LSD) and psilocybin shift patient brain dynamics closer to criticality. This proof‐of‐principle study investigates this novel therapeutic avenue and demonstrates the potential of virtual clinical trials in precision medicine.

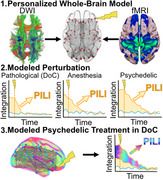

## 1 Introduction

The diverse phenomenological capacities of conscious experience are orchestrated by the emergence of spatiotemporally complex dynamics that facilitate the brain's capacity to integrate information from differentiated sources.^[^ ^1^, ^2^, ^3^ ^]^ These rich and flexible dynamics are underpinned by two key aspects of the brain^[^ ^4^, ^5^, ^6^ ^]^: its physical wiring, represented by white matter structural connectivity (SC) obtained through diffusion weighted imaging (DWI), and its neural function, approximated by the blood oxygen level dependent signal (BOLD) derived from functional magnetic resonance imaging (fMRI).^[^ ^7^ ^]^ Healthy brain dynamics are highly sensitive to perturbations and operate at a critical, or marginally subcritical regime.^[^ ^8^, ^9^, ^10^ ^]^ Criticality refers to the state at the transition point between order and disorder. At a critical point, a system develops scale‐free patterns of activity that promote long‐range communication and render it highly sensitive to perturbations. This enables maximally efficient information processing by combining adaptability with order.^[^ ^11^, ^12^, ^13^, ^14^ ^]^ At the edge between order and disorder, critical systems show the greatest perturbational sensitivity and are thought to be one of the main dynamical mechanisms through which nature produces complexity.^[^ ^15^ ^]^ Disruptions of brain function, in the presence of a healthy brain structure, can significantly alter conscious experience, such as during dreamless sleep or anesthesia. Conversely, severe brain injury, whether due to anoxia or traumatic brain injury, causes major structural damage and subsequent functional deficits, which can result in coma and post‐comatose disorders of consciousness (DoC). In states of reduced consciousness, such as DoC and anesthesia from drugs like propofol and dexmedetomidine, the brain loses its capacity to integrate information, becomes more segregated, decreases in functional complexity, and shifts away from criticality.^[^ ^16^, ^17^, ^18^, ^19^, ^20^, ^21^ ^]^ DoC includes unresponsive wakefulness syndrome (UWS) and minimally conscious state (MCS). UWS is characterized by wakefulness and reflex actions without awareness,^[^ ^22^ ^]^ while MCS involves non‐reflex behaviors (e.g., responses to command, visual pursuit) without functional communication.^[^ ^23^ ^]^

Classical psychedelics, such as lysergic acid diethylamide (LSD) and psilocybin, act upon several monoaminergic receptors. The 5‐HT2A receptor is thought to be the primary receptor responsible for the alterations in perception, mood, and numerous cognitive processes.^[^ ^24^ ^]^ Psychedelics have been proposed as promising treatments for various affective disorders in psychiatry.^[^ ^25^, ^26^, ^27^, ^28^, ^29^, ^30^ ^]^ Compared to normal wakefulness, the brain in the psychedelic state operates at an even more unstable regime, at the edge of criticality, with a higher complexity.^[^ ^31^, ^32^, ^33^, ^34^, ^35^, ^36^ ^]^ Therefore, using psychedelics to push the brain dynamics of patients with DoC from a state of pathologically low complexity closer towards criticality, and thus closer to the dynamics of a healthy brain, raises the possibility of a new treatment paradigm.^[^ ^37^, ^38^ ^]^ Such a treatment could acutely increase the conscious level of patients, potentially eliciting new conscious behaviors or enriching their phenomenological experience.

Administering psychedelics to patients with DoC presents significant challenges. Their legal status alone presents difficulties in obtaining licenses for the management and storage of materials. Additionally, administering a substance that profoundly alters phenomenological experience to a population that cannot provide consent requires comprehensive ethical considerations^[^ ^39^ ^]^ and meticulous study design. With the parameters of the model fully accessible, whole‐brain computational modeling offers a way to circumvent these challenges.^[^ ^40^ ^]^ These investigations can also provide causal mechanistic insights into structural and functional dependencies supporting the brain dynamics in different states of consciousness.^[^ ^6^, ^41^, ^42^, ^43^ ^]^

One effective way to study a dynamical system is to introduce a perturbation and observe its return to a baseline state. The perturbational complexity index (PCI) leverages this approach by calculating the algorithmic complexity of the resultant electroencephalography (EEG) signal following a transcranial magnetic current stimulation (TMS) pulse.^[^ ^44^ ^]^ Similarly, in silico perturbation protocols using whole‐brain models at the group level have been shown to distinguish between states of consciousness.^[^ ^31^, ^43^, ^45^, ^46^ ^]^ One of these approaches, the perturbative integration latency index (PILI),^31,47^ measures the amplitude and latency of the dynamical return to baseline from a modeled perturbation, thereby measuring brain dynamical stability. Increased sensitivity to a perturbation indicates a more unstable system, with dynamics that would be closer to criticality, consistent with a system with higher complexity.

The heterogeneity of functional deficiencies and structural lesions in patients with DoC makes personalized models for scientific investigation and medical applications an essential, yet aspirational target.^[^ ^40^ ^]^ In this study, we created individualized generative whole‐brain models based on Stuart‐Landau oscillators, integrating the empirical BOLD fMRI data and personalized DWI tractography data from each patient with DoC. We calculated PILI from an in silico perturbation protocol, to characterize the brain dynamics of states of consciousness, including DoC, anesthesia, and the psychedelic state. We then utilized a framework incorporating empirical LSD and psilocybin fMRI data from healthy controls to virtually simulate drug administration on each individual patient with DoC, before assessing the resulting brain dynamics using PILI. By connecting physical reality with a digital representation, we aimed to create digital twins^[^ ^47^ ^]^ of patients with DoC, and enroll them in a “phase zero” clinical trial to test the efficacy of this potentially paradigm‐shifting treatment.

## 2 Results

We built whole‐brain computational models, optimized at the individual patient level, to explore using psychedelic drugs as a treatment for DoC. We assessed brain dynamics using an in silico perturbation protocol, which measures the return to baseline dynamics after a modeled perturbation. Firstly, using group‐level models, we showed how the response to perturbation can discriminate between different states of consciousness (i.e., DoC, anesthesia from propofol, anesthesia from dexmedetomidine, and the psychedelic state under LSD and psilocybin). Then, we simulated the administration of LSD and psilocybin separately upon individualized models of patients with DoC and assessed the modeled dynamics via response to perturbation at baseline and at the simulated psychedelic state. Lastly, we characterized the empirical baseline predictive biomarkers of simulated treatment effect.

### 2.1 Response to Perturbation Differentiates between States of Consciousness

**Figure 1:**
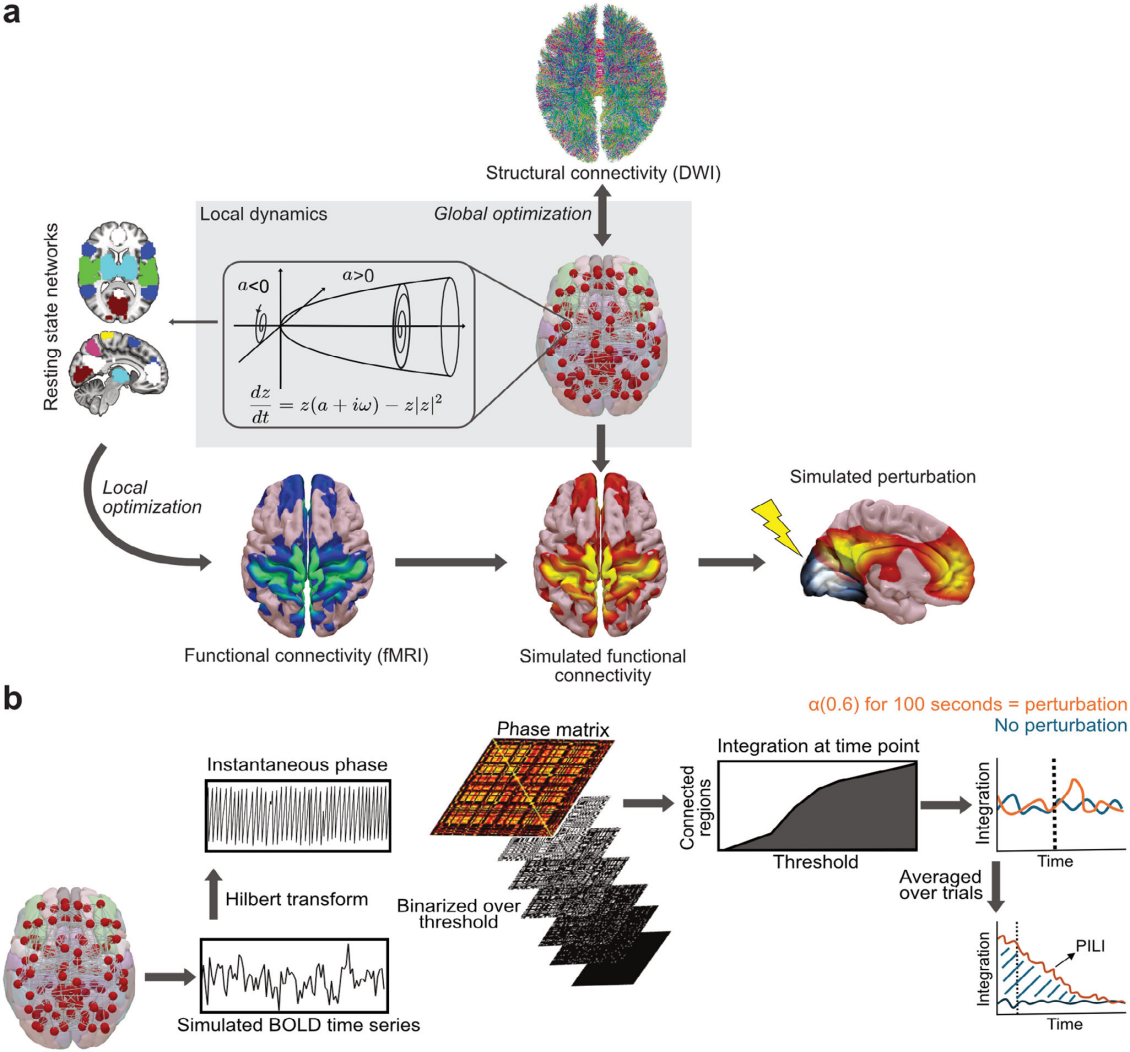
Schematic describing the main computational methods used. a) Construction of the whole‐brain models. This model uses empirical structural connectivity (SC) and functional connectivity (FC) to simulate the BOLD time series. The local dynamics are defined by a Hopf bifurcation, which depending on the value of the bifurcation parameter, can be at a stable fixed‐point (a < 0), a stable limit cycle (a > 0), and a bifurcation between both regimes (a = 0). The SC provided by white matter tractography from DWI modulates the coupling between brain regions, and the global coupling parameter scales the coupling between brain regions in the SC. The local bifurcation parameters are optimized by minimizing the Euclidean distance of the simulated FC towards the FC of the empirical fMRI data, restricted to a parameter space representing nine resting state networks obtained from an independent component analysis (one a parameter per network, with the a parameters of every region in the atlas being the linear combination of the a parameters of the networks the region belongs to). Brain dynamics are assessed by simulating a perturbation and observing the resultant return to a dynamical baseline. b) PILI protocol. We applied the Hilbert transform to the simulated BOLD signals to obtain the instantaneous phases. We constructed and binarized a phase locking matrix at each time point and calculated the number of regions in the largest connected component over thresholds. Integration was defined as the integral over all thresholds. The perturbation protocol consisted of modifying the bifurcation parameter of one brain region to the stable regime (a = 0.6) for 100 s. Integration was then calculated over 300 s in the basal unperturbed state and immediately after the model perturbation. PILI was computed as the integral between the curves of integration values over time for the perturbed dynamics (orange) compared to the maximum of the basal state dynamics (blue).

We first sought to establish an in silico perturbational biomarker that can distinguish between states of consciousness, based on the level of consciousness, and phenomenological richness of experience. This could later serve as a proxy to assess the simulated treatment response in our individual patient models. To achieve this, we created Hopf whole‐brain computational models optimized on the FC matrix obtained from the BOLD fMRI data corresponding to each state of consciousness, averaged at the group level (**Figure** 1). We used previously published data^[^ ^48^, ^49^, ^50^, ^51^, ^52^ ^]^ from several states of consciousness to create 13 different models: three for the DoC dataset (see Table S1, Supporting Information, for the demographics of the patients with DoC) (healthy controls (CNT) N = 35, UWS N = 20, and MCS N = 26), four models based on healthy subjects under anesthesia (propofol N = 13, dexmedetomidine N = 11, as well as a separate model of wakefulness for each dataset), four models based on healthy subjects in the psychedelic state (LSD N = 12, psilocybin N = 15), and the two related placebo conditions. We also performed a supplementary analysis using a model built on another psilocybin dataset, presented in Supporting Information. Briefly, each model possesses two sets of parameters: the global coupling parameter (G) and the local bifurcation parameters (a). The global coupling parameter linearly scales the coupling between brain regions in the SC matrix. The local bifurcation parameters denote the dynamics of each node: negative values produce noisy activity, while positive values lead to stable oscillatory dynamics. After retrieving the optimal global coupling value, the local parameter optimization was restricted to a nine‐parameter space representing nine resting state networks (attention, auditory, DMN, frontoparietal, limbic, sensorimotor, precuneus, thalamus, visual) defined by an independent components analysis on the fMRI data from the healthy controls of the DoC dataset (see *Model Fitting to Empirical Data* in the experimental section for more details on optimization). Optimal global parameters and local parameters for each model used in this study can be found in Tables S2 and S3 (Supporting Information).

We then investigated the dynamical properties of the different states of consciousness following an in silico perturbation. Each model was exposed to a perturbation protocol, which consisted of shifting the dynamics of one of the 90 brain regions to a more stable state for 100 s, repeated for each brain region. The integration, a measure calculated from the phase coherence synchronization between brain regions (see *Intergration* in experimental section for further details) over time was then determined immediately after the perturbation, and in a baseline state without perturbation. We then calculated PILI for each model after perturbation by summing the area beneath the perturbational integration curve until it returned to the baseline state (Figure 1b). We determined the PILI for each node by selectively perturbing one node while maintaining the dynamics of the other nodes unperturbed, in their basal state, across 100 trials (see *Model Perturbation Protocol and Perturbative Integrative Latency Index* in the experimental section for more details). These trials were then averaged to produce a single PILI value for each node. Finally, a singular, mean PILI value representing the average across all brain regions was computed.

**Figure 2:**
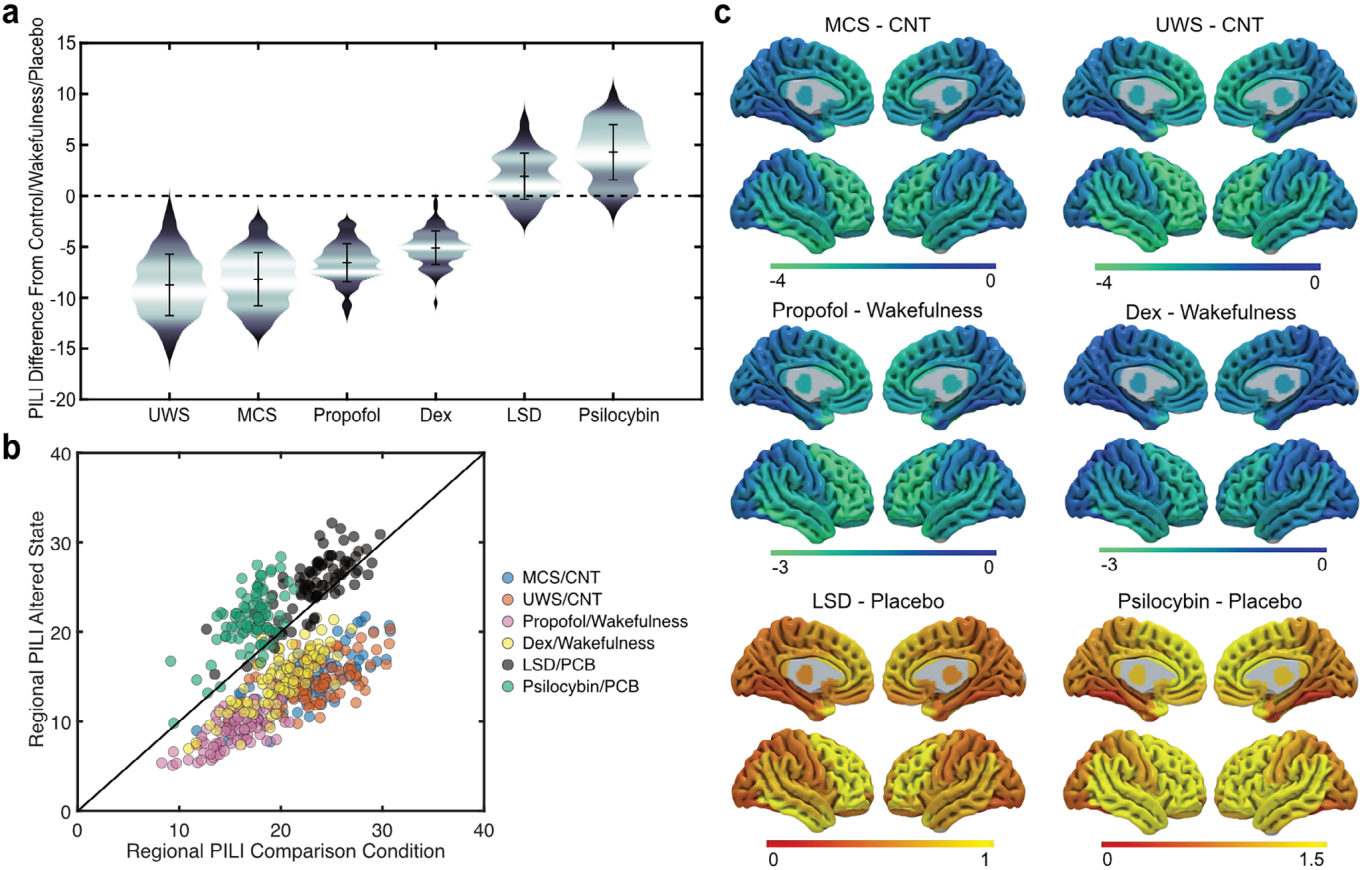
Brain dynamics assessed by PILI in whole‐brain models in different states at the group level. a) Violin plots of the distribution of PILI trials averaged over all brain regions of group‐level models in each state of consciousness. The *Y*‐axis represents the distance of each group from its respective comparison group: healthy controls for the UWS and MCS groups, the respective wakefulness conditions for propofol and dexmedetomidine, respective placebo conditions for LSD and psilocybin. b) Absolute region‐wise PILI in each state of consciousness on the *X*‐axis plotted against the respective comparison group on the *Y*‐axis. c) Absolute network‐wise changes in PILI.

To statistically compare the PILI values between states of consciousness, we took each simulation as a separate datapoint, thus having 90 brain regions and 100 simulations for each region. We calculated the Cohen's *d* effect size to compare the distributions between each condition and its associated comparison. In states of reduced consciousness, DoC patients and healthy subjects under anesthesia had decreased PILI values compared to the comparison conditions (CNT and wakefulness, for DoC and anesthesia groups, respectively) (**Figure** **2**). Specifically, UWS (Cohen's *d =* −0.52) and MCS (Cohen's *d* = −0.48) patients had lower PILI values compared with CNT. Similarly, decreases in PILI values were also observed for propofol (Cohen's *d* = −0.54) and dexmedetomidine anesthesia (Cohen's *d* = −0.32). In contrast, we found that PILI values in the psychedelic conditions were higher compared to the placebo condition, for both LSD (Cohen's *d* = 0.11) and psilocybin (Cohen's *d =* 0.28). Network‐wise changes in PILI are listed in Table S4 (Supporting Information).

**Criticality, Complexity, and Perturbations**

In dynamical‐systems theory, the critical point denotes the state that sits exactly between order and chaos. At this phase‐transition point, several interesting properties emerge. These include scale‐free distributions of activity (spanning all spatial and temporal scales), long‐range correlations, and high sensitivity to perturbations, which result in extensive and sustained responses.^[^ ^12^, ^13^, ^14^, ^15^ ^]^ The healthy brain is thought to operate in a zone near the point of criticality or marginally sub‐critical.^[^ ^8^, ^9^, ^10^ ^]^

Moving away from criticality in either direction is defined by changes in two properties of the system. Stability increases when the system shifts toward the sub‐critical, highly ordered regime. Stability is inversely related to chaos and describes how rapidly a system returns to baseline following perturbations. Highly stable (subcritical) systems produce brief, localized perturbational responses. In contrast, systems nearer to criticality show more sustained and widespread integration; thus, a greater response to perturbation. Shifting beyond the critical point into the chaotic (super‐critical) domain renders the system hypersensitive, but at the cost of losing the differentiated activity.

Complexity is inherently related to criticality. Both terms are multidimensional concepts that cannot be captured with a single metric. Complexity, as defined by Tononi et al.,^[^ ^98^ ^]^ refers to a balance between global integration and local differentiation of neural activity. Measures like Lempel‐Ziv complexity quantify some facets of this concept, but are not all‐encompassing. For example, a signal of “chaotic” noise would have a high Lempel‐Ziv complexity, despite having a suboptimal balance of differentiation and integration. Because a system at criticality simultaneously preserves local differentiation and global integration, it is also where complexity, in the sense of “integrated differentiation,” naturally peaks.

The Perturbative Integration Latency Index (PILI) captures key dimensions of criticality and complexity. PILI measures how extensively and for how long perturbation propagates through the network: smaller, transient responses reflect lower complexity (and sub‐critical states), while widespread and sustained perturbational responses indicate higher complexity and proximity to criticality.

### 2.2 Perturbations of Individualized Patient Models

We then constructed individualized whole‐brain models for each subject in our DoC dataset (20 UWS, 26 MCS, 35 CNTs). The parameters of each model were optimized towards the individual BOLD fMRI data. To define the coupling between nodes, we used the individual patient SC, generated by white matter tractography from the DWI data. From this point onwards, all the analyses were performed using these digital twins of each subject.

**Figure 3:**
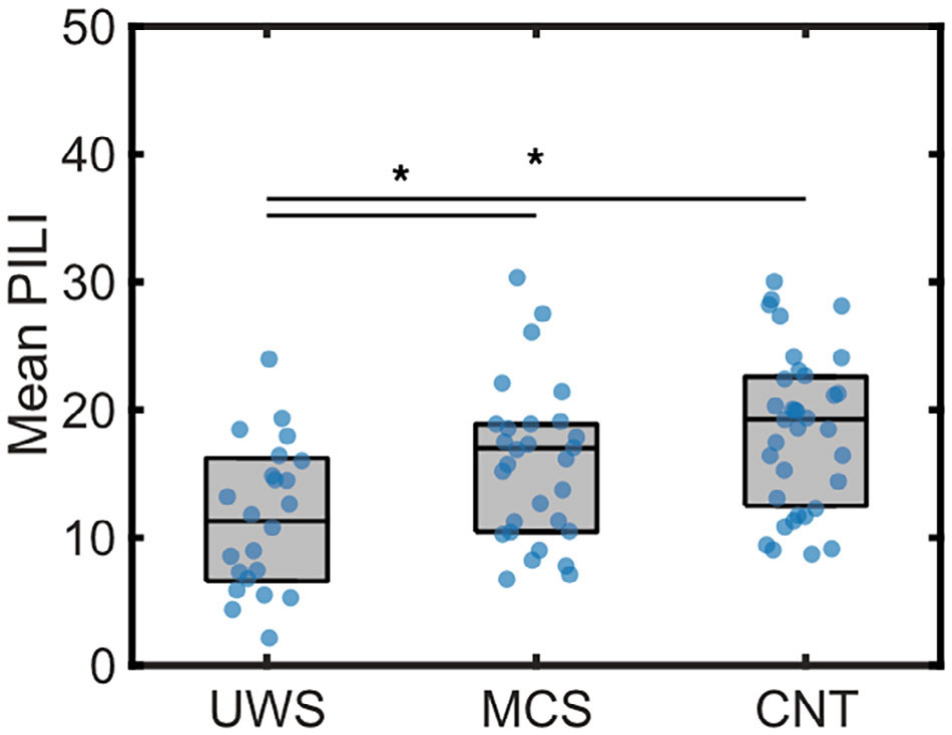
Brain dynamics assessed by PILI in personalized whole‐brain models of individual patients with DoC and controls. Each blue dot represents the PILI value from a patient or control. Stars represent significance at *p* < 0.05 from the Tukey's HSD test following a one‐way ANOVA.

We performed the perturbation protocol on each patient individually and calculated PILI values for each of the 90 brain regions. In all our analyses on individual models, we decided to consider a single PILI value in each brain region as the mean across all 100 simulations. The results from a one‐way ANOVA between the PILI values across MCS patients, UWS patients, and CNT showed a significant main effect of group (F(2, 78) = 8.84). Post‐hoc comparisons using Tukey's honestly significant difference (HSD) test indicated that PILI values were higher in the CNT group compared to the UWS group (mean difference = 6.80, 95% CI [2.94, 10.66], *p* < 0.001). Additionally, PILI values were higher in the MCS group compared to the UWS (mean difference = 4.11, 95% CI [0.09, 8.12], *p* = 0.044). There was no significant difference between the CNT and MCS groups (mean difference = 2.69, 95% CI [−0.92, 6.30], *p* = 0.182) (**Figure** **3**). All reported *p*‐values were corrected for multiple comparisons.

### 2.3 Simulation of LSD and Psilocybin on Patients with DoC: A Virtual Clinical Trial

**Figure 4:**
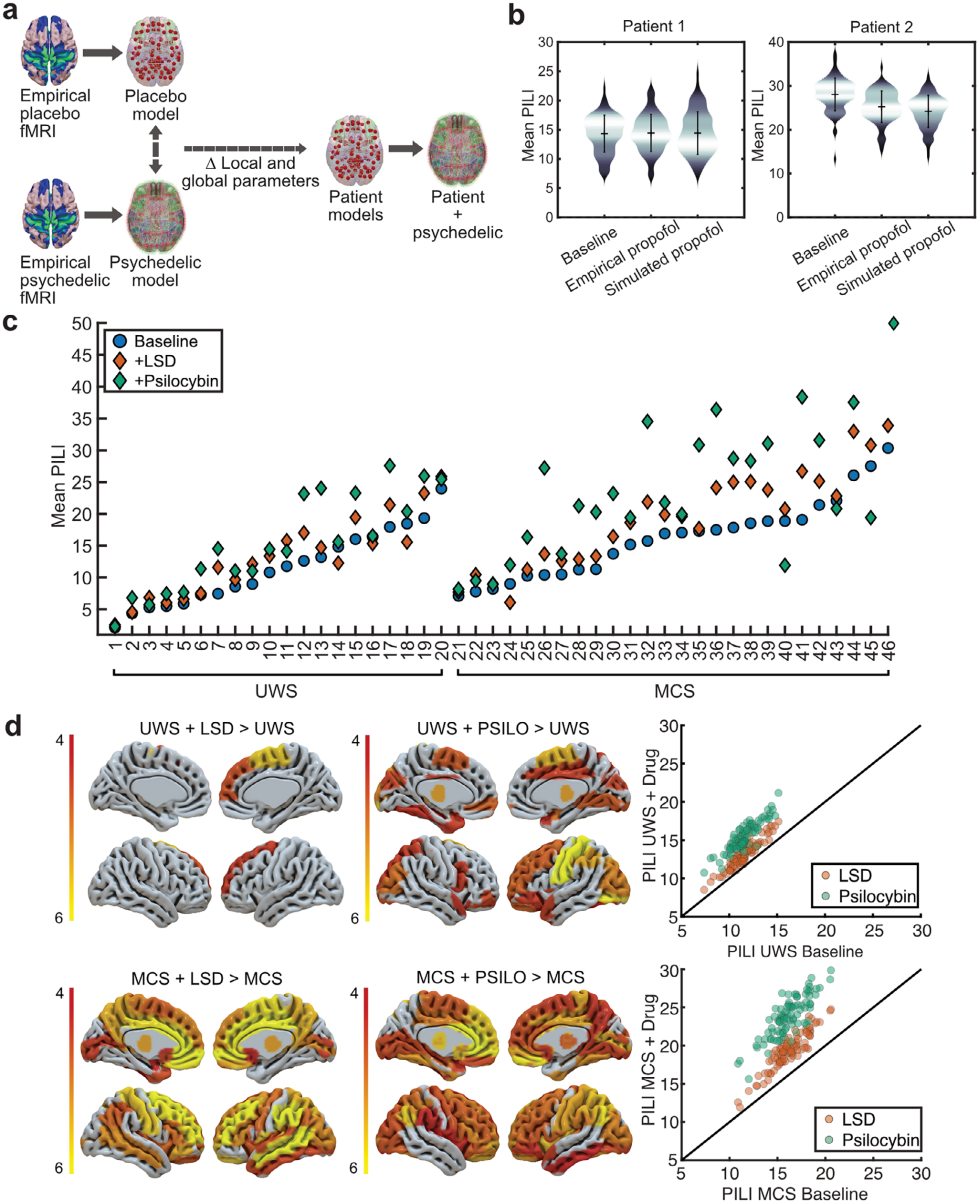
Brain dynamics assessed by PILI in personalized whole‐brain models of individual patients with DoC before and after the simulation of LSD and psilocybin. a) The virtual pharmacology method. Computational models of each state of consciousness in the same subjects are constructed. The global coupling and local bifurcation parameters are extracted and applied to the models built on individual patients to simulate the administration of the drug, this shift in parameters is enacted before performing the PILI perturbational protocols. b) Validation of the virtual pharmacology approach using an independent set of two patients receiving a light dose of propofol. Baseline and empirical propofol show the PILI values before and after receiving propofol. Simulated propofol shows the PILI values after simulating propofol. c) Individual patient models before and after simulation of LSD and psilocybin. Blue circles represent each patient at baseline, orange diamonds represent LSD, and green diamonds represent psilocybin. d) Left: Plotted *t*‐statistic values of region‐wise *t*‐tests displaying brain regions with significant increases, in PILI from simulating LSD and psilocybin in patients with UWS (top) and MCS patients (bottom), Bonferroni corrected for multiple comparisons for the 90 brain regions. Right: Average PILI values for each of the 90 brain regions, averaged across patients at the baseline state (*X*‐axis) and after the simulation of LSD (orange) and psilocybin (green) (*Y*‐axis).

The next steps were to simulate the administration of LSD and psilocybin on the individual patient models. This was achieved through developing a virtual pharmacology method (see *Virtual Pharmacology Approach* in the Experimental Section for more details). Briefly, this involved creating whole‐brain models optimized at the group level using the BOLD fMRI data from healthy individuals being administered a drug and at the respective placebo or comparison conditions. For each drug condition individually, we extracted the changes in the global coupling and local bifurcation parameters that manifested upon comparing the placebo/comparison model to the drug model. We then applied the shift in parameters representing the virtual administration of the drug separately to each patient's model (**Figure** **4**a). The parameter changes representing the simulation of LSD and psilocybin can be seen in Table S5. The resulting stimulatory drug‐induced changes in brain dynamics were then characterized by calculating PILI at the baseline state (parameters in their optimized values for that specific patient) and after the simulation of the drug (after shifting the parameters that represent the drug effects).

While direct data from DoC patients under psychedelics are unavailable, we sought to provide validation for our virtual pharmacology framework using data from propofol anesthesia. We had access to two unique cases in which BOLD fMRI data were acquired before and during the administration of a light dose of propofol (average effect site concentration 1.80 µg mL^−1^). Our propofol dataset in healthy individuals had two levels of propofol dose: a light dose of propofol (1.75 µg mL^−1^ average blood plasma concentration), resulting in a Ramsay score of 3, and a moderate dose (3.20 µg mL^−1^ average blood plasma concentration), resulting in an unresponsive state (Ramsay score of 5/6). Therefore, we set out to validate our model of a virtual administration of LSD and psilocybin by simulating a virtual administration of a light dose of propofol and comparing the resulting simulated brain dynamics to the models built on the empirical data of the patient under a light dose of propofol. Using our virtual pharmacology approach, we optimized models based on empirical BOLD fMRI for normal wakefulness and a light dose of propofol and extracted the changes in parameters between these conditions before applying them to the individual patient models. We then assessed the brain dynamics through PILI at the baseline, after empirical propofol, and after the simulation of propofol (Figure 4b). The mean differences in residual per‐region PILI between the simulation‐derived model and empirical‐derived model were 0.0002 in one patient and around 1 in another. One patient with a low baseline PILI had a relatively small effect from the simulation of propofol, whereas another patient with a relatively high baseline PILI had a somewhat larger decrease in PILI as a result of propofol. The simulation of propofol using our virtual pharmacology method seems to capture these differential effects. Please consult Supporting Information and Figure S2 (Supporting Information) for a longer discussion about the validity of our virtual pharmacology approach.

Subsequently, we simulated the administration of LSD and psilocybin to each individual patient model. Simulating LSD significantly increased the PILI values of patients with DoC, with a greater effect on patients in the MCS (z = 4.13, *p *< 0.001) compared to patients with UWS (z = 2.8, *p *< 0.001). This was also the case after simulating psilocybin, as the increases in PILI values observed in patients with UWS (z = 3.71, *p *< 0.001) were lower than those observed in patients in the MCS (z = 3.92, *p *< 0.001). Next, we examined how the global response to perturbation differed across initial stimulation site, calculating the PILI values in each region, averaged across subjects at the baseline, and after simulating LSD and psilocybin. To better contextualize the regional changes in PILI, we calculated the differences in PILI in the nine resting state networks, which define our local parameter space (Table S6). In the MCS group, after the simulation of both LSD and psilocybin, the networks with the largest differences in PILI values were the DMN, the attention, and the frontoparietal network. In the UWS group, the sensorimotor network had the highest increase in PILI values for both the LSD and psilocybin conditions. For both LSD and psilocybin, region‐wise PILI values did not correlate with regional SC after Bonferroni correction for the four comparisons (LSD: MCS r = 0.21, *p* = 0.04, UWS r = 0.05 *p* = 0.56; psilocybin: MCS r = 0.13, *p* = 0.23; UWS r = 0.02, *p* = 0.84), nor FC (LSD: MCS r = 0.17, *p* = 0.11, UWS r = 0.12, *p* = 0.84; psilocybin: MCS *p* = 0.26, r = 0.02, UWS r = 0.05, *p* = 0.61).

### 2.4 Baseline Predictors of Treatment Response in Individual Patients

We then sought to identify which structural and functional capacities of the brains of patients with DoC at baseline were associated with a high simulated treatment effect. In this way, we could ascertain the most promising candidates for potential psychedelic treatment. We defined the simulated treatment effect to be the difference in PILI between the baseline state and after the simulation of LSD and psilocybin. Within each of the MCS patients, UWS patients, and the associated control group, we quantified the strength of FC across the nine resting state networks that define our local bifurcation parameter space. Additionally, we computed several graph theory metrics from the patients' SC matrices, including global efficiency, local efficiency, centrality, and graph strength. We also determined the mean FA values from DWI data. See Tables S7 and S8 (Supporting Information) for statistical analyses of the structural and functional capacities at baseline.

**Figure 5:**
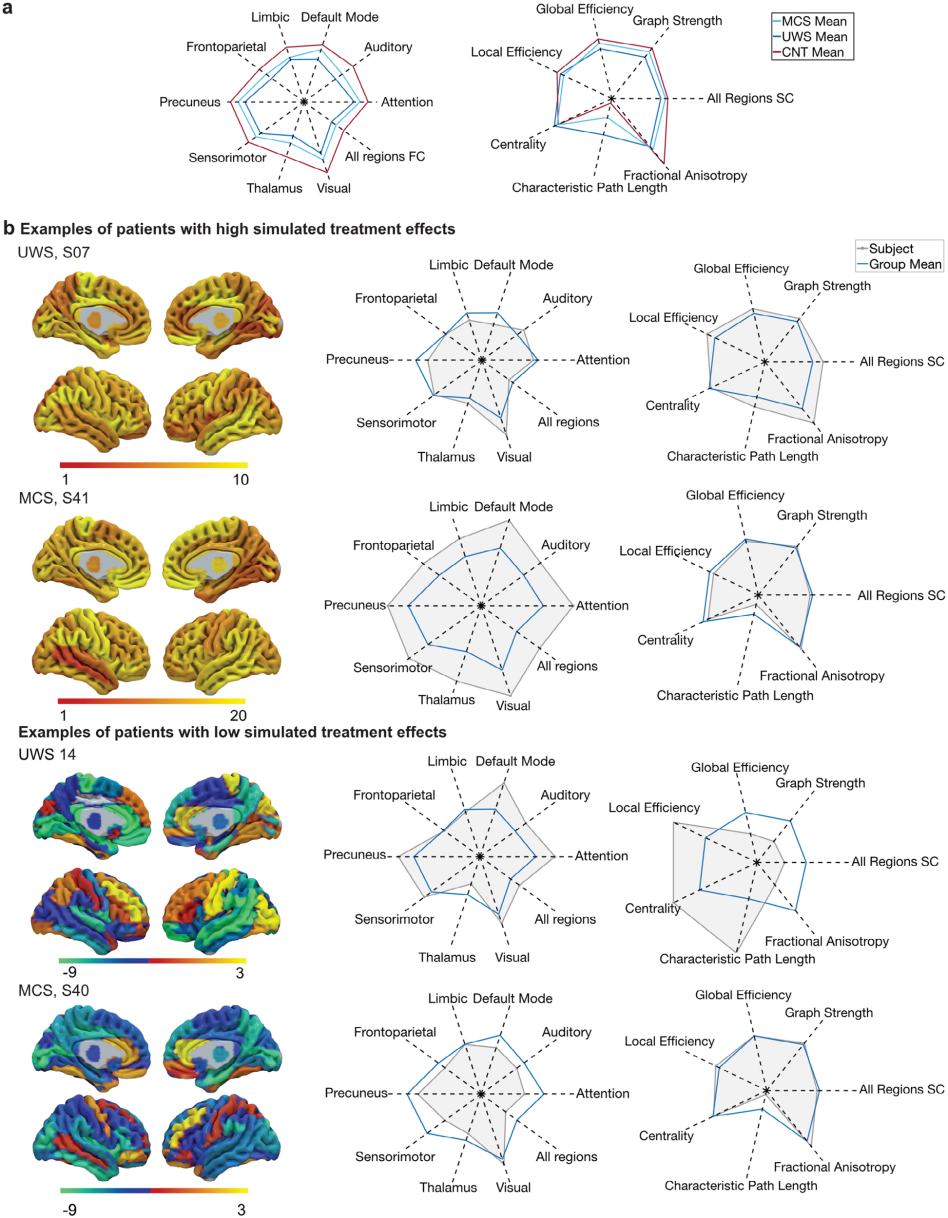
Structural and functional connectivity characteristics at baseline in the patients with DoC. a) Group average patient and healthy control graph theory structural metrics and functional connectivity in resting state networks at baseline. b) Examples of patients and their functional and structural capacities at baseline. One example is chosen from each diagnostic group that underwent the highest and lowest average simulated treatment effect between psilocybin and LSD conditions. Left: Absolute regional changes in PILI values for each subject. Warmer colors (red and yellow) indicate higher PILI changes, while cooler colors (green and blue) indicate lower PILI changes. Right: Radar plots comparing the functional and structural capacities of each patient (filled grey shape) to the mean of the associated diagnostic group (blue line). The left radar plot illustrates the average FC across all regions and within the networks that define the local parameter space: frontoparietal, default mode, auditory, attention, visual, thalamus, sensorimotor, precuneus, and limbic networks. The right radar plot shows average SC metrics across all regions, and the average global efficiency, graph strength, local efficiency, betweenness centrality, fractional anisotropy, and characteristic path length.

Here, we present four representative example patients and their structural and functional characteristics at baseline to elucidate insights into which characteristics could predict treatment efficacy (**Figure** **5**b). These include one patient from each diagnostic group who had the largest increases and decreases in PILI, averaged between psilocybin and LSD. MCS S41 showed the largest increase in PILI values for both LSD and psilocybin. At baseline, MCS S41 had one of the highest mean FC strengths of the group (z = 4.46, *p *< 0.001). However, their mean SC strength was not significantly different from the group mean (*z* = 0.4, *p* = 0.74). UWS S07 had one of the highest simulated treatment effects of the group. This patient had a mean FC which was not significantly different from the group mean (*z* = 0.4291, *p* = 0.70). However, the mean SC was significantly higher than the group mean (*z* = 3.16, *p* = 0.002). Examining those subjects with the worst simulated treatment outcome, UWS S14 had a relatively high FC (*z* = 2.65, *p* = 0.008), yet one of the worst SC of the group (*z* = −4.20, *p *< 0.001). Whilst MCS S40 had a SC not significantly different from the mean of the group (*z* = 0.79, *p* = 0.43), yet a low mean FC (*z* = −2.98, p = 0.002).

**Figure 6:**
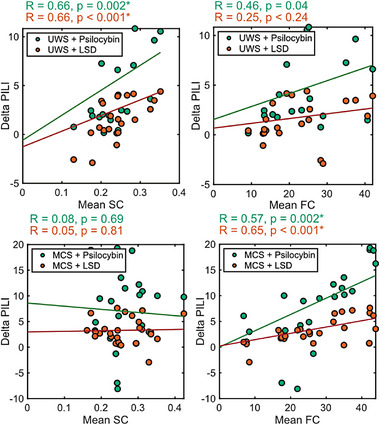
Scatter plots showing correlations between the changes in PILI values as a result of simulating LSD and psilocybin (Delta PILI) and the average baseline structural connectivity (SC) and functional connectivity (FC) in UWS patients (upper) and MCS patients (lower). Regression lines are indicated in orange (LSD) and green (psilocybin). Associated correlation coefficients (R) and *p*‐values are stated above in orange (LSD) and green (psilocybin). Star indicates significance after Bonferroni correction for the eight correlations.

To further investigate these relationships and identify baseline biomarkers of predicted treatment efficacy, we performed correlation analyses between each structural and functional capacity and the simulated treatment effect. In the UWS group, only the mean SC (LSD: *r* = 0.66, *p* = 0.001; psilocybin: *r* = 0.66, *p *< 0.002) correlated with the simulated treatment effect and remained significant after Bonferroni correction (**Figure** **6**). In the MCS group, there were no significant correlations between structural capacities and the simulated treatment effect for either drug (Table S9, Supporting Information). However, the mean FC strongly correlated with the simulated treatment effect for both LSD (r = 0.65, *p *< 0.001) and psilocybin (*r* = 0.57, *p* = 0.002) in the MCS group. Additionally, in both the LSD and psilocybin conditions, FC in the DMN significantly correlated with the simulated treatment effect, and for LSD, FC in the attention, frontoparietal network, and sensorimotor network also correlated with the simulated treatment effect (Table S10, Supporting Information).

## 3 Discussion

We used individualized whole‐brain computational models in patients with DoC based on empirical DWI and fMRI data. Using each digital twin, we conducted a virtual clinical trial to explore a novel treatment paradigm: using psychedelic drugs, specifically LSD and psilocybin, to increase the complexity of brain activity in patients with DoC. To this end, we first set out to validate PILI, a modeling‐based perturbational biomarker based on the integration of a modeled perturbation in distinguishing between states of consciousness, namely, DoC, anesthesia, and the psychedelic state. Second, we simulated the administration of LSD and psilocybin on each patient and assessed the resulting brain dynamics as a proxy for simulated treatment response. Third, we characterized the baseline predictive biomarkers associated with increases in PILI, ultimately identifying predictive biomarkers of potential treatment efficacy.

Assessing the brain's response to a perturbation allows us to understand how it integrates external information and provides insights into to the criticality of brain dynamics and their implied complexity. Our results showed that PILI could distinguish between states of consciousness according to the phenomenological richness of experience. States of diminished consciousness, such as DoC (UWS and MCS) and anesthesia (propofol and dexmedetomidine), showed lower PILI values compared to the control subjects and wakefulness conditions, respectively. States with a higher richness of experience, ergo the psychedelic state (LSD and psilocybin), possessed higher PILI values compared to their placebo conditions. This supports research linking the complexity of brain activity and criticality to consciousness,^[^ ^53^ ^]^ ideas which are at the core of the integrated information theory of consciousness.^[^ ^54^, ^55^ ^]^ States in which consciousness is diminished, such as in DoC or anesthesia, possess more stable brain dynamics, with lower complexity.^[^ ^16^, ^17^, ^20^, ^21^, ^36^, ^56^ ^]^ This dynamic rigidity, manifesting through a faster return to the baseline following a perturbation, exemplifies the shift towards a sub‐critical regime. Support from TMS‐EEG studies on anesthesia and DoC shows lower evoked complexity in these states compared to normal wakefulness.^[^ ^44^ ^]^ During the psychedelic state, the increase in PILI epitomizes the brain's shift even closer to criticality. This echoes several previous lines of investigation showing that the psychedelic state is associated with an increase in brain complexity, an increased repertoire of available states, and more critical dynamics.^[^ ^31^, ^32^, ^33^, ^34^, ^35^, ^36^ ^]^ Both propofol and dexmedetomidine were administered at unresponsive doses. However, dexmedetomidine often induces dreaming, which occurs less often with propofol.^[^ ^57^ ^]^ This likely contributes to dexmedetomidine's intermediate PILI value and underscores the importance of considering phenomenological differences between unresponsive states.^[^ ^58^ ^]^

When comparing PILI values from diagnostic groups at the single‐subject level, there was a difference in mean PILI values between the UWS and MCS groups, but not between the MCS and CNT groups. This mirrors findings from studies on the PCI, where, below a numerical cut‐off, a patient is considered unconscious, and above this level, there is less discrimination between conscious states like MCS and CNT.^[^ ^44^, ^59^ ^]^ It also aligns with clinical assessments of consciousness at the bedside, where UWS patients do not show any signs of consciousness, yet MCS patients exhibit signs of residual consciousness. The established threshold allows PCI to function as a diagnostic tool at the single‐subject level. While PILI currently lacks this discriminative capacity at the single‐subject level, future developments in computational modeling should aim to create a diagnostic tool of comparable precision.

We also developed a method to administer virtual pharmacological treatments based on extracting the changes in parameters observed due to the drug effects and applying them to models that represent other states of consciousness, here, patients with DoC. In principle, this approach could be generalized to extract parameter changes that represent any pharmacological or neuromodulatory intervention that acutely results in changes in brain connectivity, such as anesthetics, stimulation techniques, or even simulating other conditions, such as listening to music. These changes could then be applied to any model at the group, or single‐subject level to simulate such conditions. Crucially, the success of this method depends on the parameter extraction originating from a dataset that estimates each state within the same subjects. This ensures the most accurate estimation of the treatment effects by imposing the specificity of functional and structural changes. To illustrate the importance of this, we present (Figure S3, Supporting Information) and discuss (Supporting Information) analyses using a separate fMRI dataset from healthy subjects under psilocybin, acquired with a between‐subjects design. Here, the PILI was slightly lower in the psilocybin condition compared to the placebo condition, and the simulated treatment response in patients with UWS was not associated with the SC.

The PILI increases in response to the administration of LSD and psilocybin to patients with DoC. This represents a shift in brain dynamics towards criticality, with greater effects observed in the MCS compared to UWS groups. The simulated psychedelic‐induced changes in brain dynamics in MCS patients show a similar absolute shift towards criticality as those seen between UWS and CNT, and between anesthesia and wakefulness. Whilst at face value, this may imply that the absolute size of the change in complexity could be enough to support a conscious state, the phenomenological implications and behavioral consequences of these changes in PILI remain an open question. It is conceivable that the administration of a psychedelic to patients with DoC may invoke a supraphysiological state, which does not improve consciousness. Predicting such phenomenological effects is outside of the remit of this study, and early clinical work, such as a recently published case study, can begin to approach these questions.^[^ ^60^ ^]^

In patients in the MCS, after both LSD and psilocybin, some of the largest network‐wise increases in PILI were observed in the DMN, attention, and frontoparietal. Notably, the DMN and frontoparietal networks also showed the largest absolute increases in PILI in group‐level models of LSD and psilocybin in healthy subjects. Each of these higher‐order networks is associated with supporting facets of consciousness and cognition and shows diminished connectivity in patients with DoC. Particularly, patients with DoC show diminished DMN connectivity, with patients in the MCS having stronger DMN connectivity compared to patients with UWS.^[^ ^61^, ^62^ ^]^ Therefore, alterations in the dynamics within these networks suggest that they may impart some changes in experience. Interestingly, in patients in the MCS, the DMN, frontoparietal, and attention networks showed the highest correlation between baseline connectivity and the simulated treatment effect. This implies that the strong connectivity in these networks at baseline is necessary to support the functional and dynamical changes entailed by the administration of a psychedelic drug, thus any potential treatment response. In the UWS group, the only network with significant changes in PILI for both psilocybin and LSD was the sensorimotor network. This is likely due to the large cortical structural damage present in other networks, restricting the propagation of activity into such areas. The lower‐order role of the sensorimotor network in initiating and coordinating movements rather than being associated with consciousness per se, decreases the likelihood of treatment outcomes positively augmenting consciousness and cognition in patients in the UWS.

Whilst generally consistent, psilocybin and LSD had some differential effects, with psilocybin showing more variability and a greater effect in patients with UWS. Although both drugs produce similar phenomenological effects,^[^ ^63^ ^]^ they have distinct pharmacological profiles: LSD targets D1–3 receptors, while psilocin, the active metabolite of psilocybin, inhibits the serotonin transporter.^[^ ^64^ ^]^ Psilocybin and LSD have been shown to have unique patterns of functional reorganization dependent on distinct neurotransmitter mechanisms.^[^ ^65^ ^]^ Also, recent modeling work simulating the stimulation of different receptors in patients with DoC has demonstrated the differential effects these receptors have on brain dynamics. Compared to 5‐HT2A receptor stimulation, activation of D1 and D2 receptors had a minimal impact on the modeled trajectory of brain dynamics in patients with DoC towards that of controls.^[^ ^66^ ^]^ These differences in pharmacology and connectivity likely contribute to the variations in their simulated effects on brain dynamics and highlight their potential differing utility in treatments.

In patients with UWS, the SC was correlated with the simulated treatment response, whereas in patients in the MCS, it was the FC that was predictive. Neither the network FC connectivity, nor average FC correlated with simulated treatment response in patients with UWS, while in patients in the MCS, none of the structural characteristics correlated with treatment response. Despite there being no significant difference in mean SC between MCS and UWS patients, characteristic path length  was significantly lower in patients with UWS compared to patients in the MCS. Additionally, global efficiency and average graph strength were significantly different between controls and patients with UWS, yet not between controls and patients in the MCS. Graph strength refers to the sum of connection weights linked to each node. Global efficiency reflects the average inverse shortest path length between all node pairs, while characteristic path length is the average shortest path length across the network. Each of these metrics reflects different aspects of network efficiency. Reductions in connectivity within hub‐regions would result in decreases across these measures. Thus, decreases in efficiency measures without a significant drop in mean SC may indicate regional disconnection, particularly affecting hub regions, while maintaining connectivity or even overcompensating in other regions in patients with UWS. Since efficient organization is already severely impaired inpatients with UWS, the primary limiting factor for enabling a dynamic response to psychedelics becomes whether any structural scaffolding remains at all, rather than how efficiently it is organized. In contrast, in MCS patients, sufficient structural efficiency is preserved, such that the total strength of SC becomes less important. Once a minimal level of efficiency of communication is maintained, FC rather than SC becomes the primary determinant of capacity for psychedelic‐induced changes in complexity. Our results point to a hierarchical relationship between structure, function, and complexity. Efficient white‑matter architecture provides the first‐layer scaffold that allows spatially differentiated regions to exchange information, upon which richer functional dynamics can emerge. If that scaffold falls below a minimum level of efficiency, complexity cannot be supported. Consistent with this view, mean SC predicted treatment outcome in UWS patients more strongly than any specific graph‑theoretic metric. In practical terms, topological details of network efficiency contribute relatively little once damage is extensive, since the sheer number of intact white‑matter fibers cap a patient's capacity for complex brain activity. This implies a bleak prognosis for patients with UWS whose hub regions have suffered major structural damage. However, there is a growing interest in another use case of psychedelics in those with severe brain injury that seeks to harness the neuroplastic effects to augment recovery immediately in the acute phase of brain injury.^[^ ^67^ ^]^ Future work utilizing models that account for plasticity could explore this. Taken together, our correlational analyses suggest that a patient in the MCS with high average FC and specifically within the DMN and attention networks, has the greatest likelihood to have the largest changes in brain dynamics following a psychedelic drug.

In silico experiments with personalized generative whole‐brain computational models hold substantial potential for mechanistic investigations, drug discovery, and ultimately personalized medicine. Creating a unique digital twin of each patient offers a powerful methodological approach for simulating pharmacological treatments or neuromodulation techniques, without exposing patients to actual risks, in effect creating a virtual clinical trial.^[^ ^40^ ^]^ The considerable heterogeneity in the etiology, lesion site, and symptoms of patients with DoC makes this approach particularly appealing, simulating interventions and subsequently tailoring them for each patient with respect to drug choice, dosage, stimulation type, etc. Notable progress has already been made in epilepsy research, where personalized models have provided a valuable means to test stimulations and simulate post‐surgical outcomes.^[^ ^68^, ^69^ ^]^ As computational resources grow and methodologies refine, digital twins are poised to become highly valuable in clinical neuroscience, enabling personalized treatment strategies, and providing mechanistic insights.

This study has several limitations. The inherent range in PILI values within each subject at the single‐subject level underscores the limited applicability of PILI for between‐subject comparisons, emphasizing its more appropriate use for characterizing states within the same subjects. Similarly, differences in acquisition protocols and scanning parameters make comparing PILI values between datasets challenging, as evidenced by the variability in PILI values across different control group models (see Figure S4 and Supporting Information). The Hopf model is a phenomenological model that does not aim to replicate underlying biology. However, recent studies have shown that many complex bottom‐up models at the regional level merely replicate a Hopf bifurcation.^[^ ^70^ ^]^ Therefore, while the Hopf model is limited in its mechanistic explanatory power compared to biophysical models, it still similarly captures regional and global brain dynamics. This work relies on the superposition assumption, being that a linear subtraction of the effects of psychedelics on controls well predicts the action of the drugs in patients with DoC. Rather than a uniform shift in the local/global parameters models, it is likely that patients with DoC have a different response to psychedelics compared to healthy individuals. Despite this significant assumption, it provides a useful approximation in the absence of empirical data on patients under a psychedelic. Future empirical data may be used to refine these parameter sets for patients with DoC. Additionally, our method is based on parameter estimations from a single dataset, which could have bias.

Here, we ran a virtual clinical trial by simulating the administration of LSD and psilocybin in individualized whole‐brain models of patients with DoC. We showed that psychedelics increase the brain's response to perturbation and globally bring the brain dynamics of patients with DoC closer to criticality. We also reveal that patients in the MCS are more likely to increase in complexity compared to patients with UWS and that this is dependent upon the strength of the baseline FC. This work builds on the idea of using psychedelics for DoC and contributes toward computational personalized medicine and drug discovery.

## 4 Experimental Section

### Dataset Descriptions

**Figure 7:**
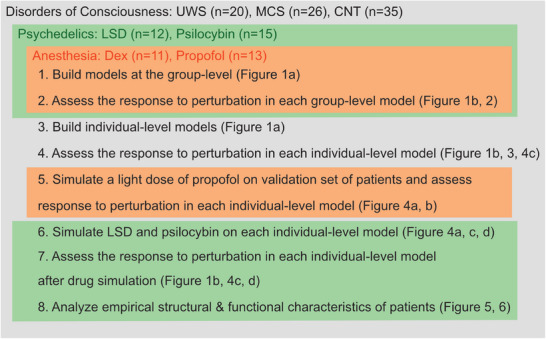
Schematic overview of each step of the analyses and where each dataset was used. Steps covered by orange indicate those where the anesthesia dataset was used. Steps covered by green indicate those where the psychedelic datasets were used. Steps covered by grey indicate those where the Disorders of Consciousness datasets were used. Dex: Dexmedetomidine. Results of the between‐subjects psilocybin dataset are presented in the Supporting Information. Unresponsive wakefulness syndrome (UWS), minimally conscious state (MCS), healthy controls (CNT).

This study included previously published fMRI data from six datasets, three from the University and University Hospital of Liège (DoC, propofol, dexmedetomidine), two from Imperial College London (LSD, psilocybin), and one from Maastricht University (psilocybin). For the DoC dataset, DWI acquired in the same patients and healthy controls was also used. An auxiliary dataset of DWI and fMRI data from two patients was also used: One UWS and one MCS patient, before and after they received a light dose of propofol. See Dataset Descriptions in the Supporting Information for further details of the acquisition and demographic information. **Figure** **7** briefly describes each step of analysis in the study and where each dataset was used. The DoC, propofol, and dexmedetomidine datasets received approval from the Ethics Committee of the Faculty of Medicine at the University of Liège (DoC: 2009/241, propofol: 2007/191, dexmedetomidine: 2012/135). Written informed consent was obtained from the healthy participants and, in the case of patients with DoC, from their legal representatives. The LSD and psilocybin studies from Imperial College London were approved by the UK National Health Service Research Ethics Committees in West‐London (LSD: 13/LO/0229) and Bristol (psilocybin: 08/H0101/144), respectively. All participants provided informed consent. The psilocybin study from Maastricht University obtained ethical approval from Maastricht University's Medical Ethics Committee (NL60352.068.17 / METC173006). Informed consent was obtained from all participants.

### Functional Magnetic Resonance Imaging and Functional Connectivity Estimation

Since the datasets were acquired at different locations, we endeavored to mitigate scanner and acquisition differences by re‐preprocessing all data with the same pipeline and following uniform denoising procedures. The preprocessing of fMRI images was conducted utilizing FSL (FMRIB Software Library v6.0, Analysis Group, FMRIB, Oxford, UK). Structural T1‐weighted images were processed using the fsl_anat function, which involved brain extraction, bias field correction, and normalization. Functional MRI data underwent preprocessing with MELODIC (Multivariate Exploratory Linear Optimized Decomposition into Independent Components). The Maastricht psilocybin fMRI data uniquely underwent realignment and susceptibility distortion correction with FSL_topup to correct for magnetic field distortion caused by the high‐strength 7T field. Main preprocessing steps for all datasets included discarding the initial five volumes, motion correction with MCFLIRT, brain extraction using BET (Brain Extraction Tool), spatial smoothing with a 5 mm FWHM Gaussian kernel, rigid body registration, and applying a high‐pass filter with a cutoff of 100.0 s. Additionally, single‐session ICA with automatic dimensionality estimation was performed in order to identify noise‐driven components. Each component was visually inspected using the FSLeyes package in Melodic mode to classify the single‐subject independent components as either signal or noise/lesion‐driven artifacts, based on the spatial map, time series, and temporal power spectrum. Noise components were then regressed out using fsl_regfilt to obtain a cleaned version of the functional data. Furthermore, BOLD data were detrended, demeaned, and band‐pass filtered between 0.01 and 0.08 Hz. To obtain the BOLD time series for the 90 cortical and subcortical brain regions defined by the AAL atlas (excluding the cerebellum), FSL tools were used in each individual's native EPI space. This parcellation was found to be particularly suitable for studying whole‐brain spatiotemporal dynamics.^[^ ^31^, ^45^, ^71^ ^]^ Given that the computational models were personalized at the individual level, utilizing 90 regions provided a balanced approach between spatial resolution and computational feasibility. The cleaned functional data were registered to the T1‐weighted structural image using FLIRT. The T1‐weighted image was then normalized to the standard MNI space using FLIRT (12 DOF) and FNIRT. The resulting transformations were concatenated, inverted, and applied to warp the resting‐state atlas from MNI space to the cleaned functional data. Nearest‐neighbor interpolation ensured the preservation of labels. The BOLD time series for each of the 90 brain regions was extracted for each subject in their native space using fslmaths to create a binary mask of each brain region and fslmeants to obtain the time series of each mask. Each of the 90 regional average BOLD signals was then filtered in the 0.04–0.07 Hz range, which has previously been used due to its functional relevance and resistance to noise.^[^ ^72^, ^73^, ^74^, ^75^ ^]^ Pairwise Pearson correlation coefficients were then computed between all 90 brain regions for each subject. For the group‐level matrices, the Fisher transform was applied to the *r*‐values to derive *z*‐values for the 90×90 functional connectivity (FC) matrices before being averaged and subsequently back‐transformed.

### Anatomical Connectivity

The patient structural connectomes were created using advanced, single‐subject analyses of the diffusion MRI (dMRI) dataset of patients with DoC, including 35 age‐matched controls. MRI scans were performed using a Siemens 3T Trio scanner (Siemens Inc., Munich, Germany) with a 64‐channel head coil. Using tools from MRtrix3 https://mrtrix.org/, raw dMRI data were corrected,^[^ ^76^, ^77^ ^]^ denoised, and corrected for Gibbs ringing artifacts,^[^ ^78^ ^]^ as well as distortions induced by motion, eddy currents, and EPI/susceptibility artefacts.^[^ ^79^, ^80^ ^]^ The response functions from the pre‐processed diffusion‐weighted images were estimated using the Dhollander algorithm and used to calculate fiber orientation distributions (FODs) via constrained spherical deconvolution (CSD).^[^ ^81^ ^]^ Here, MRtrix3Tissue (v5.2.8; https://3tissue.github.io) was utilized, a variant of MRtrix3,^[^ ^82^ ^]^ which enabled three‐tissue constrained spherical deconvolution, generating separate FODs for white matter (WM), grey matter (GM), and cerebrospinal fluid (CSF) SS3T‐CSD,^[^ ^83^ ^]^ from single‐shell (+b = 0) diffusion MRI data. Whole‐brain tractography was then performed,^[^ ^84^ ^]^ generating 20 million streamlines per subject^[^ ^85^ ^]^ using dynamic seeding. The spherically informed filtering of tractograms (SIFT2) algorithm was applied to align the fiber density of the reconstructed streamlines with the underlying white matter structures.^[^ ^84^, ^86^ ^]^ Compared to tractograms constructed solely by the number of streamlines, SIFT2 adjusted the weight of individual streamlines to ensure alignment with the underlying image data.^[^ ^84^, ^86^, ^87^ ^]^ SIFT2 is highly reproducible^[^ ^88^ ^]^ and enhances the biological interpretability of the estimated white matter tracts.^[^ ^89^ ^]^ Probabilistic tractography was then performed using the iFOD2 (improved 2nd order integration over fiber orientation distributions) algorithm, which enhanced anatomical plausibility. During tracking, the direction for each step was obtained by sampling from the FOD at the current position, with the probability of a particular direction being proportional to the FOD amplitude. Additional tractography settings included a default step size of 0.8 mm, a maximum angle between successive steps of 45°, a maximum length of 250 mm, a minimum length of 5 mm, a cutoff fractional anisotropy (FA) value of 0.08, and the use of *b*‐vectors and *b*‐values from the diffusion‐weighted gradient scheme in FSL format. Concurrently, the b0 image in native space was registered to the T1 anatomical image using FSL FLIRT.^[^ ^90^, ^91^ ^]^ The T1 structural image was then normalized to standard space using FLIRT and FNIRT. The resulting transformations were reversed and applied to the AAL atlas in standard MNI space using a nearest neighbor algorithm to convert the atlas to each subject's native space. The number of streamlines connecting each pair of regions was then estimated to produce the final SC matrix for each patient. To summarize, for each participant, a 90×90 symmetric weighted network of SC was constructed, which represented the density of white matter tracts between anatomical regions of the brain. The FA values were generated by firstly estimating the diffusion kurtosis tensor map from the pre‐processed DWI image. A map of tensor‐derived FA for each patient was then averaged across voxels to produce one FA value per subject. Graph theory metrics were calculated using The Brain Connectivity Toolbox^[^ ^92^ ^]^ implemented in MATLAB (version R2017a, MathWorks, Natick, MA). Graph strength, global efficiency, characteristic path length, average local efficiency, and betweenness centrality were calculated to capture different aspects of network structure. These measures provided insights into node importance, communication efficiency, and overall network topology.

### Computational Whole‐Brain Model

A whole‐brain model was used to simulate the brain activity of each of the 90 cortical and subcortical brain regions in the AAL atlas. In particular, the dynamics of each region were described by means of coupled Stuart–Landau oscillators in the normal form of supercritical Hopf bifurcations.^[^ ^31^, ^43^, ^45^, ^46^ ^]^ This model simulated spontaneous signals akin to the average BOLD signal in a given brain region. The coupling between oscillators representing each region was driven by a given SC. The model at node *j* was described by the following set of coupled differential equations:

(1) $$\frac{dz_{j}}{dt}=z_{j}\;\left(a_{j}+iw_{j}-{\left|z_{j}\right|}^{2}\right)+G\sum_{k=1}^{N}C_{jk}\left(z_{k}-z_{j}\right)+\beta \eta_{j\;}$$

where *z* is a complex variable *z_j_* = *x_j_*  + *iy_j_*, *w_j_* is the natural node frequency (estimated as the average peak frequency of each region of the BOLD recordings of each group or subject, for the group and single‐subject models, respectively), *η*_*j*_is additive Gaussian noise, *β* is the scaling factor for the Gaussian noise (set to 0.04), *C* is the SC matrix, where *C_jk_* is the coupling between nodes *j* and *k*, *G* is the global coupling that acts as a scaling factor for the SC matrix for all nodes, and $a_{j}$ is the bifurcation parameter controlling the behaviour of the oscillator. For  a≈0  the system is at a supercritical Hopf bifurcation, and the Gaussian noise induces complex dynamics as the system oscillates between both sides of the bifurcation. For a<0, the system decays to a stable fixed point at *z_j_* =  0 and noise‐induce oscillations appear due to the Gaussian noise. For a>0,  the system dynamics set into a stable limit cycle with a frequency of $f_{j}=\frac{\omega_{j}}{2\pi }$. The SC matrix was scaled to a maximum value of 0.2 to ensure oscillatory dynamics bifurcations.^[^ ^31^, ^43^, ^45^, ^46^ ^]^ The fMRI BOLD signal simulation was obtained as the real part of *z* (*x_j_*). The stochastic differential equations were solved by means of the Euler–Maruyama method.

For all the group models involving healthy subjects, including those for psychedelics, anesthesia, and the associated wakefulness and placebo comparisons, the SC matrix representing the average of the healthy controls in the DoC dataset was used. This approach was necessary as the authors did not have access to the individual SC data for each subject in each dataset. In previous modeling research, a template SC obtained from a commonly used dataset was usually employed, which represented the white matter connectivity of healthy subjects. This was assumed to well represent the SC of healthy controls in general.^[^ ^31^, ^42^, ^43^, ^45^, ^46^, ^93^ ^]^ Here, the same approach was adopted by using the SC estimate from the healthy control group of the DoC dataset. This ensured that the strength of the connectivity values, and thus the optimal parameters for the models employing a healthy structure, were in the same range as those of patients with DoC.

Crucially, models of patients with DoC included the empirical structural data for each patient. The diverse etiology of patients with DoC often resulted in significant structural differences between patients. By using personalized SC, the inherent bias of assuming a healthy white matter structure in DoC patients was avoided, which enabled building more robust models incorporating more empirical information. Individualized single‐subject whole‐brain models were created using the individual structural and functional data.

### Model Fitting to Empirical Data

This work involved the creation of seven models at the group level and 46 models at the single‐subject level. For the group‐level analyses, the FC averaged over each subject in each condition was used to obtain the parameters for each group model. For the single‐subject analyses, the individual FC of each patient was used to fit their parameters. Two parameters were fitted to the models in two steps. First, the global coupling *G* was fitted to the FC matrix by exploring a parameter range in steps of 0.01 starting from 0 until the fitting curve reached an optimum value. Three hundred seconds of BOLD activity were simulated each time. The same filtering applied to the empirical data was applied to the simulated time series, and their FC matrices were obtained. Afterwards, *G* was optimized based on the Kolmogorov–Smirnov distance (KS distance) between the empirical and simulated FC. For each *G* value, 500 simulations were performed, and the curve of the average KS distance was used to determine the optimal *G* value at the local minimum.

Using the KS distance, the distribution of the FC values of the simulations was brought to a similar level to that of the empirical data. After fixing the *G* values for each group/subject as a starting point, in a second step, the a values were also fitted in order to fine‐tune their dynamics by adjusting the bifurcation parameters of the regions. For each region *j*, the bifurcation parameter $a_{j}$ was obtained as the linear combination of nine resting state networks including that region. These resting state networks were obtained by means of a group independent component analysis (ICA) of the fMRI dataset from the healthy controls in the DoC dataset. This was implemented through the MELODIC ICA functionality of FSL, with automatic dimensionality estimation. Each component was manually inspected and identified as either noise or a resting state network. This resulted in the identification of nine resting state networks. These were then named according to their similarity with canonical resting state networks (default mode [DMN], auditory, attention, visual, thalamus, sensorimotor, precuneus, frontoparietal, limbic). Where there was not a corresponding canonical resting state network, the network was named based on the brain areas involved, e.g., thalamus. These IC maps were then thresholded and binarized to produce masks. Each network mask was then compared to the AAL atlas to see which regions overlapped with each component mask at the voxel level. This produced network components in the AAL space. The IC maps and corresponding AAL expression can be seen in Figure S1 (Supporting Information). Thus, the parameter space was reduced to a dimension of 9 (one for each network). The Euclidean distance was used as the goodness of fit parameter to optimize the fit for each region, and a genetic algorithm to find the optimal values of the *a* parameters of each network as in refs. [43, 94]. Nine initial values (one for each network) were simulated, the default parameters of the MATLAB function ga were used, the algorithm was run ten times, and the average *a* values of the networks over the course of the ten runs were used as the final optimal value. Ten was chosen as a balance between computational power requirements and finding the true optimal value. For each network, the parameter range of its associated *a* values to explore by the genetic algorithm was set to −0.2 to 0.2.^[^ ^43^ ^]^

### Integration

Integration refers to the capacity of the brain to assimilate communication between discrete processing units. The integration measure used in this study evaluates the synchronization of brain activity measured by the BOLD signal and was previously defined in refs. [31, 45]. The Hilbert transform was applied to filtered BOLD signals to extract the instantaneous phase φ_*j*_(*t*) for each region *j*. The Hilbert transform derives the analytic representation of a real‐valued signal, in this case, the BOLD time series. The synchronisation between pairs of brain regions was characterized as the difference between their instantaneous phases. At each time point, the phase difference P_jk_(t) between two regions *j* and *k* was calculated as

(2) $$P_{\mathrm{jk}}\;\left(t\right)=e^{-i\left|\varphi_{j}\left(t\right)-\varphi_{k}\left(t\right)\right|}$$

Here, P_jk_ (t) =  1 when the two regions are in phase, *φ*_j_ (t) =  *φ*_k_(t) (full synchronized). At any time *t*, the phase interaction matrix P(t) represents the instantaneous phase synchrony of each pair of brain regions. The phase interaction matrix was then binarized over 100 evenly spaced thresholds between 0 and 1. Then, the size of the largest connected component across all brain regions was obtained at each threshold, thus creating a function of connected components as a function of the threshold. The integration at time *t* was then denoted as the integral of this curve (Figure 1b). The integration measure reflected the synchronization of brain region dynamics at different thresholds. This reflected communication at all possible scales afforded by the atlas, including long‐range communication, a hallmark of criticality. A larger connected component signified extensive synchronization across brain regions, with enhanced capacity for flexible, long‐range communication. Conversely, smaller components reflected localized, less integrated dynamics.

### Model Perturbation Protocol and Perturbative Integrative Latency Index

The perturbation protocol has been previously described in refs. [31, 45]. Briefly, the local bifurcation parameters of the Hopf model were used to simulate a perturbation through switching each a from its original base value to a more synchronous regime (*a* = 0.6) for 100 s. This value was based on previous studies employing the PILI^[^ ^31^, ^45^ ^]^ and has been shown to well differentiate conscious states. The integration was computed over 400 s of simulated BOLD time series in the basal state and immediately after the perturbation offset. This protocol was performed at every brain region in the 90 AAL parcellation and repeated 100 times. After perturbation, the bifurcation parameter was reset to its original value obtained from the model fitting procedure. Plotting the integration at each time point in the perturbed state created a curve showing how the integration changes over time in response to a perturbation; the basal integration line represented the integration in the absence of any perturbation, with the alpha parameters kept at their optimized values. PILI in a particular region was calculated as the area under the perturbational integration curve from perturbation offset to the interception point with the basal state line of integration. This was repeated for each brain region for 100 trials before being averaged to obtain a regional PILI value. The singular mean PILI represented the average PILI over all brain regions. Since the PILI was based on the integral of the integration curve, it characterized both the strength and latency of the return to its basal state after a model perturbation. PILI quantified how extensively perturbations propagate through the network. A small response to perturbation reflected lower complexity, whereas a large response to perturbation indicated widespread integration of multiple regions, consistent with higher complexity and a system closer to criticality.^[^ ^31^, ^42^, ^45^, ^94^ ^]^ Slowness of recovery to perturbation is a key property of critical systems, where it is sometimes referred to in the literature as “critical slowing”.^[^ ^95^ ^]^ A biological mechanism underlying increases in the local bifurcation parameter was suggested to be akin to increasing the local excitability.^[^ ^96^, ^97^ ^]^ With this extension, such kinds of perturbations resembled the effects of those seen with transient increases in cortical excitability induced experimentally by methods like anodal transcranial direct current stimulation^[^ ^97^ ^]^

### Virtual Pharmacology Approach

To simulate the administration of psilocybin and LSD to the individualized patient models, a virtual pharmacology method was developed. This was based upon group‐level models optimized to the BOLD fMRI data from healthy individuals being administered a drug (LSD, psilocybin) and at the respective placebo conditions. To simulate the administration of the target drug, the global coupling parameters and local bifurcation parameters representing each condition were then extracted and compared within each condition to attain a shift in the parameters that represented that particular condition. This change in parameters thus represented a set of instructions on how to shift the model dynamics from one condition to another. This shift was then applied in parameters representing the virtual administration of the drug (LSD and psilocybin) separately to each patient's model. As a validation step, the shift in parameters was also extracted from group models optimized to the BOLD fMRI data from healthy individuals before and after a light dose of propofol, and a virtual administration of propofol in two patients was simulated. The effect of modeled brain dynamics after the virtual administration of propofol would then be compared with modeled brain dynamics from these two unique cases, where BOLD fMRI data before and after the administration of a light dose of propofol were present.

### Statistical Analyses

When comparing the mean PILI values between whole‐brain models built at the group level, Cohen's *d* effect size was used to compare the distributions of PILI values produced by each model. Since each group level model can in theory, produce an infinite number of simulations, thus artificially inflating the sample size, any *p*‐values produced by frequentist statistical tests were less applicable in these cases.

For all analyses using individual models, a standard frequentist approach was employed since each model essentially can act as a unique datapoint with its own distribution, akin to a subject. To compare the PILI values between diagnostic groups, from the personalized models of DoC patients, Mann–Whitney *U* tests were performed. To compare the mean PILI values obtained from simulating the psychedelic drug to the baseline PILI values, Wilcoxon signed‐rank tests were performed. Paired *t*‐tests were used to evaluate the region‐wise changes in PILI as a result of simulating the psychedelic with Bonferroni correction for the 90 regions. Wilcoxon signed rank tests were used to compare the network‐wise PILI values between the baseline state and simulated drug state with Bonferroni correction for each of the nine resting state networks.

## Conflict of Interest

VB has a financial relationship with Orion Pharma, Edwards Medical, Medtronic, Gnenthal, and Elsevier.

## Supporting information



Supporting Information

## Data Availability

All requests for raw and analyzed data and materials are reviewed by the chief investigator of each respective original work: O.G. for the patients with DoC data, R.L.C.‐H. for the LSD and psilocybin (within subjects) data, J.G.R. for the psilocybin (between subjects) data, and VB for the anesthesia data. All data needed to evaluate the conclusions in the paper are present in the paper and/or Supplementary Information.
