# Supplementary material for: A Virtual Clinical Trial of Psychedelics to Treat Patients With Disorders of Consciousness
Source: Adv Sci (Weinh). 2025 Nov 20;13(11):e11780. doi: 10.1002/advs.202511780 (PMC12931207; doi:10.1002/advs.202511780)
Supplement: Supplementary file 1 — Supporting Information [file ADVS-13-e11780-s001.pdf]

Supplementary Materials

A virtual clinical trial of psychedelics to treat patients with disorders of consciousness

Naji L.N. Alnagger, Paolo Cardone, Charlotte Martial, Yonatan Sanz Perl, Iván Mindlin, Jacobo D Sitt, Leor Roseman, Robin Carhart-Harris, David Nutt, Pablo Mallaroni, Natasha Mason, Johannes G Ramaekers, Vincent Bonhomme, Steven Laureys, Gustavo Deco, Olivia Gosseries*, Pablo Núñez*, Jitka Annen*

* Contributed equally

Olivia Gosseries
Email: ogosseries@uliege.be

This file includes:

Supporting text
Figures S1 to S4
Tables S1 to S10
Supplementary References

Supporting Text

Dataset Descriptions

This study includes fMRI data from 6 datasets, 3 from the University and University Hospital of Liège (DoC, propofol, dexmedetomidine), 2 from Imperial College London (LSD, psilocybin) and 1 from Maastricht University (psilocybin). For the DoC dataset, we also used diffusion weighted imaging (DWI) images, acquired in the same patients and healthy controls.

<u>DoC dataset</u>

The study received approval from the Ethics Committee of the Faculty of Medicine at the University of Liège. Written informed consent was obtained from the healthy control participants and from the legal representatives of the patients. We included 46 adult patients with DoC, comprising 26 in MCS (6 females, age range 23–74 years; mean age ± SD, 47 ± 17 years), and 20 in UWS (6 females, age range 31–74 years; mean age ± SD, 52 ± 16 years), along with 35 gender-matched healthy controls (14 females, age range 19–72 years; mean age ± SD, 40 ± 14 years). Data for the DoC patients were recorded 57.98 ± 203.65 months post-injury. There are 10 acute patients with DoC (4 UWS, 5 MCS) in the sample. We also used data from an additional two patients (1 UWS and 1 MCS) in which two functional magnetic resonance imaging (fMRI) sequences were acquired, before and immediately after the administration of a light dose of propofol (average effect site concentration 1.80 µg/mL). The diagnosis for DoC patients was confirmed through repeated behavioural assessments using the Coma Recovery Scale-Revised (CRS-R) conducted by trained staff, which evaluates auditory, visual, motor, sensorimotor function, communication, and arousal[1]. DoC patients were included if their MRI exams were conducted without anaesthetic sedation and if they had undergone at least five CRS-R assessments within a 14-day period with one assessment conducted the same day as the MRI acquisition. Patients were excluded based on the following criteria: (i) having any significant neurological, neurosurgical, or psychiatric disorders prior to the brain injury leading to DoC, (ii) having contraindications to MRI such as implanted electronic devices or external ventricular drains, and (iii) being medically unstable or having extensive focal brain damage, defined as damage affecting more than two-thirds of one hemisphere. Further details on the demographics and clinical characteristics of the patients are provided in Supplementary Table 1. Structural and functional MRI (fMRI) data were acquired on a Siemens 3T Trio scanner (Siemens Inc, Munich, Germany). The BOLD fMRI resting state (i.e. task free) was acquired using EPI, gradient echo with following parameters: volumes = 300, TR = 2000 ms, TE = 30 ms, flip angle = 78∘, voxel size = 3 × 3 × 3 mm3, FOV = 192 × 192 mm2, 32 transversal slices, with a duration of 10 minutes. Subsequently, structural 3D T1-weighted MP-RAGE images were acquired with following parameters: 120 transversal slices, TR = 2300 ms, voxel size = 1.0 × 1.0 × 1.2 mm3, flip angle = 9∘, FOV = 256 × 256 mm2). The diffusion MRI (dMRI) data were collected using a single echo planar imaging sequence gradient scheme with 64 non-collinear gradient directions (total acquisition time [TA] = 14:47 min, b = 1,000 s/mm^2, field of view [FOV] = 256 mm^2, voxel size = 2.0 mm isotropic, repetition time [TR] = 9,700 ms, echo time [TE] = 92 ms).

<u>Propofol and dexmedetomidine datasets</u>

The propofol and dexmedetomidine datasets have been previously published[2,3]. Both datasets have identical experimental protocols and received approval from the Ethics Committee of the Medical School of the University of Liege (University Hospital, Liege, Belgium). After receiving informed consent from the volunteers, thirteen healthy control subjects (age range 18-31 years) underwent propofol-induced sedation. Eleven healthy control subjects (age range 19-29 years) underwent dexmedetomidine-induced sedation. fMRI data were collected during normal wakefulness with eyes

closed and during propofol/dexmedetomidine-induced sedation. Propofol/dexmedetomidine was infused through an intravenous catheter placed in a vein of the right hand or forearm, and an arterial catheter was placed in the left radial artery. Sedation was achieved using a computer-controlled intravenous infusion of propofol to maintain constant effect-site concentrations (for details on the procedure, see[2]). The drug plasma and effect-site concentrations were estimated using a three-compartment pharmacokinetic model. After reaching the appropriate effect-site concentration, a 5-minute equilibration period was allowed to ensure equilibration of drug distribution between compartments. Arterial blood samples were taken immediately before and after the scan in each clinical state for subsequent determination of the drug concentration. The level of consciousness was clinically evaluated throughout the study using the Ramsay scale [4]. Responsiveness was assessed through volitional hand squeezing in response to a verbal command from the experimenter. If there was no response to the command (Ramsay 5–6), the subject was considered to be in deep sedation. If the response to verbal command was clear but slow (Ramsay 3), they were in considered to be in mild sedation. One resting state fMRI sequence was acquired at mild sedation and deep sedation. Propofol plasma concentration was 1.75μg/mL for mild sedation and 3.2μg/mL for deep sedation (unresponsiveness). For each assessment of consciousness level, the Ramsay scale verbal commands were repeated twice. Before and after each scanning session, a reaction time task was also performed to provide additional information on the clinical state of the volunteers. The healthy subjects had no MRI contraindications, no history of neurological or psychiatric disorders, and no drug consumption that could significantly affect brain function. The propofol and dexmedetomidine datasets were acquired on a 3T Siemens Allegra scanner (Siemens AG, Munich, Germany). The fMRI resting-state scans were acquired using the following parameters: EPI, gradient echo, volumes = 200; TR = 2460 ms, TE = 40 ms, voxel size = 3.45 × 3.45 × 3 mm^3, FOV = 220 × 220 mm, 32 transverse slices, 64 × 64 × 32 matrix size. The structural images were acquired using 3D T1-weighted MP-RAGE with the following parameters: 120 transverse slices, TR = 2250 ms, TE = 2.99 ms, voxel size = 1 mm^3, flip angle = 9°, FOV = 256 × 240 × 160 mm.

LSD dataset

We used previously published data fully described here [5]. Briefly, 12 healthy participants were scanned under six different conditions: LSD resting state, placebo resting state, LSD and placebo resting state while listening to music, and LSD and placebo resting state after listening to music. LSD and placebo sessions were separated by at least 14 days, with the condition order balanced across participants, who were blinded to this order. For this study, we only used the LSD resting state and placebo resting state data. All participants provided informed consent. The experimental protocol was approved by the UK National Health Service Research Ethics Committee, West-London. The experiments conformed to the revised Declaration of Helsinki, the International Committee on Harmonisation Good Clinical Practice guidelines, and the National Health Service Research Governance Framework. Each participant received either 75 μg of LSD (intravenous, I.V.) or saline/placebo (I.V.) 70 minutes prior to MRI scanning. Participants reported noticing subjective drug effects between 5- and 15-minutes post-dosing. The drug effects peaked between 60- and 90-minutes post-dosing. The subsequent plateau of drug effects varied among individuals, generally lasting for four hours post-dosing. MRI acquisition started approximately 70 minutes post-dosing and lasted about 60 minutes. After each of the three scans, participants performed subjective ratings inside the scanner via a response box. The scans conducted with saline/placebo were considered baseline MRI scans compared to the LSD scans. Neuroimaging data were collected using a 3T GE HDx MRI system. Data were recorded using a gradient echo-planar

imaging sequence, TR/TE = 2000/35 ms, field of view = 220 mm, 64 × 64 acquisition matrix, parallel acceleration factor = 2, 90° flip angle.

Psilocybin Imperial dataset

We used previously published data described here[6]. Briefly, 15 healthy volunteers participated in two MRI scanning sessions separated by at least 14 days. Participants were at least 21 years old, with no personal or family history of major psychiatric disorders, no substance dependence, no cardiovascular disease, and no history of adverse responses to psychedelic drugs. All subjects had prior experience with psilocybin but had not used it within 6 weeks of the study. The study received approval from a National Health Service research ethics committee, and informed consent was obtained from all participants. During each session, participants received either psilocybin (2 mg dissolved in 10 mL saline, administered via a 60-second intravenous injection) or a placebo (10 mL saline, administered via a 60-second intravenous injection) in a counterbalanced design. Infusions commenced precisely 6 minutes after the start of the 12-minute fMRI scans. Psilocybin's effects were immediate and persisted throughout the scanning session. Neuroimaging data were collected using a 3T GE HDx MRI system. Anatomical scans preceded functional scans and were thus performed before drug or placebo administration. Structural scans were acquired using 3D fast spoiled gradient echo sequences in an axial orientation with a field of view of 256 × 256 × 192 and a matrix of 256 × 256 × 192, yielding 1 mm isotropic voxel resolution (repetition time/echo time TR/TE = 7.9/3.0 ms; inversion time = 450 ms; flip angle = 20). BOLD-weighted fMRI data were acquired at 3T using a gradient echo EPI sequence with TR/TE = 3000/35 ms, a field of view of 192 mm, a 64 × 64 acquisition matrix, a parallel acceleration factor of 2, and a 90° flip angle. Fifty-three oblique axial slices were acquired in an interleaved fashion, each 3 mm thick with no slice gap (3 × 3 × 3-mm voxels). Following the same motion exclusion criteria as the LSD dataset, nine subjects (seven men, mean age 32 ± 8.9 years) were included in the analysis.

Psilocybin Maastricht dataset

We analyzed previously published data, described in detail here[7]. Briefly, data from 49 healthy volunteers with previous experience with a psychedelic drug not within the 3 months prior to the study. Following a randomized, placebo-controlled, double-blind parallel group design, participants were assigned to one of two conditions (0.17 mg/kg psilocybin, or placebo) such that groups were matched for age, sex, and educational level. The drug was administered orally in a closed cup containing bitter lemon (placebo) or bitter lemon and psilocybin (powder). Informed consent was obtained from all subjects. The study obtained ethical approval from the Maastricht University's Medical Ethics Committee and was in accordance with the Medical Research Involving Human Subjects Act (WMO) as well as with the code of ethics on human experimentation from the declaration of Helsinki. Following criteria outlined in the original publication, one subject was excluded due to having a maximum framewise displacement of >0.75mm (half-voxel size). Four subjects were excluded on the basis of <5 minutes of the scan remaining after scrubbing. One further subject was excluded due to missing BOLD values in several brain regions. The final sample was 22 in the psilocybin group and 26 subjects in the placebo group. Participants underwent structural MRI 50-min post psilocybin/placebo administration, in addition to 6-min resting-state fMRI 102-min post psilocybin/placebo administration during the peak subjective drug effects. All images were acquired in a MAGNETOM 7T MRI scanner using the following acquisition parameters: TR = 1,400 ms; TE = 21 ms; field of view = 198 mm; flip angle = 60°; oblique acquisition orientation; interleaved slice acquisition; 72 slices; slice thickness = 1.5 mm; voxel size = 1.5 mm, isotropic. Furthermore, participants were presented with a black fixation cross on

a white background during the scanning session and were asked to focus on the cross, clear their minds, and lie as still as possible.

Model Validation

To validate our virtual pharmacology approach, we performed some additional perturbational analyses. Firstly, we demonstrated the importance of the distinct distribution of local parameters in dictating the dynamics of the model. Instead of applying the changes in bifurcation parameters which represent the difference between the drug state and the placebo state, each of the nine bifurcation parameters in each single patient model corresponding to each of the functional networks was modified by a random addition between -0.3 and +0.3. These random values were generated from a uniform distribution using the rand function in MATLAB. The rationale of this was to show that an arbitrary change in the local bifurcation parameters would not result in increases in PILI like those seen when simulating LSD or psilocybin. We then calculated PILI in each patient at baseline, and after a random shift in alpha parameters. In almost all cases, shifting the alpha parameters randomly, resulted in decreases in PILI values (Figure S2a). This displays the vital importance of local bifurcation parameters in maintaining the complex dynamics of these models. We also simulated the administration of LSD and psilocybin on 35 single subject models optimized to our healthy control BOLD fMRI and DWI dataset (Figure S2b). We showed that almost all subjects have increases in PILI from simulating LSD and psilocybin. Also, as with the group level models based on the empirical data, the simulation of psilocybin had a greater effect than LSD. Furthermore, we created a group-level model optimized to the BOLD fMRI placebo condition and then applied the changes in local and global parameters extracted from the LSD and PCB models (Figure S2c). The rationale behind this was to compare the PILI values obtained from the group level model with that obtained from simulating the LSD using our virtual pharmacology method. The simulation of LSD on the placebo model resulted in a mean PILI value of 25.47, similar to the modest increase in PILI of 25.21, obtained directly from the LSD model. This evidences the methods capacity to simulate the drug condition.

Supplementary discussion:

The inherent range in PILI values within each subject at the single-subject level highlights the its more appropriate use for characterizing states within the same subjects. The differences between acquisition protocols and scanning parameters mean that comparing PILI values between datasets is challenging, in spite of taking steps to mitigate this via applying the same preprocessing pipelines in each dataset. This is evident from the variability in PILI values across different control group models (Figure S4) and within individual control subjects (Figure 4a and Figure S2b). We also performed a supplementary analysis of psilocybin with a between-subject design to further illustrate this. The PILI values in the psilocybin group compared to the placebo group showed a slight deviation towards decreases in the psilocybin group (Cohens d = -0.029) (Figure S3a). Similarly, the regional distribution of PILI values indicates that most values tend to cluster around the zero-line or a slight negative deviation, indicating no difference between conditions (Figure S3b).The absence of a difference in PILI between psilocybin and placebo at the group level is likely due to the fact that different subjects who received psilocybin may have different baseline PILI values. We already observe that PILI can vary across single-subject models of healthy controls, with values ranging from 10 to 30 (Figure S2b). Therefore, the range of PILI values at a baseline between two groups (i.e., those receiving psilocybin and those receiving placebo) may have influenced the absence of increases in PILI seen in this dataset. Furthermore, when estimating the treatment effect, using different subjects for each condition means that both SC and FC

changed between conditions. In a within-subject design, only FC changes as a result of drug administration. Although, we can generally assume that within some bounds, all healthy subjects have similar SC, the specificity of the virtual pharmacology method, which uses changes in local bifurcation parameters to estimate changes in brain functional dynamics likely benefits from estimating this change within the same subjects, thus keeping the structure constant. This suboptimal estimation of treatment effects for this specific use case likely underlies the absence of correlation between the baseline SC and changes in PILI as a result of simulating psilocybin in patients with UWS, which were strongly correlated in the other two psychedelic datasets. Therefore, this dataset demonstrates the need for subject specificity both in comparing the brain dynamics between conditions and when estimating the treatment effect through extracting parameter sets.

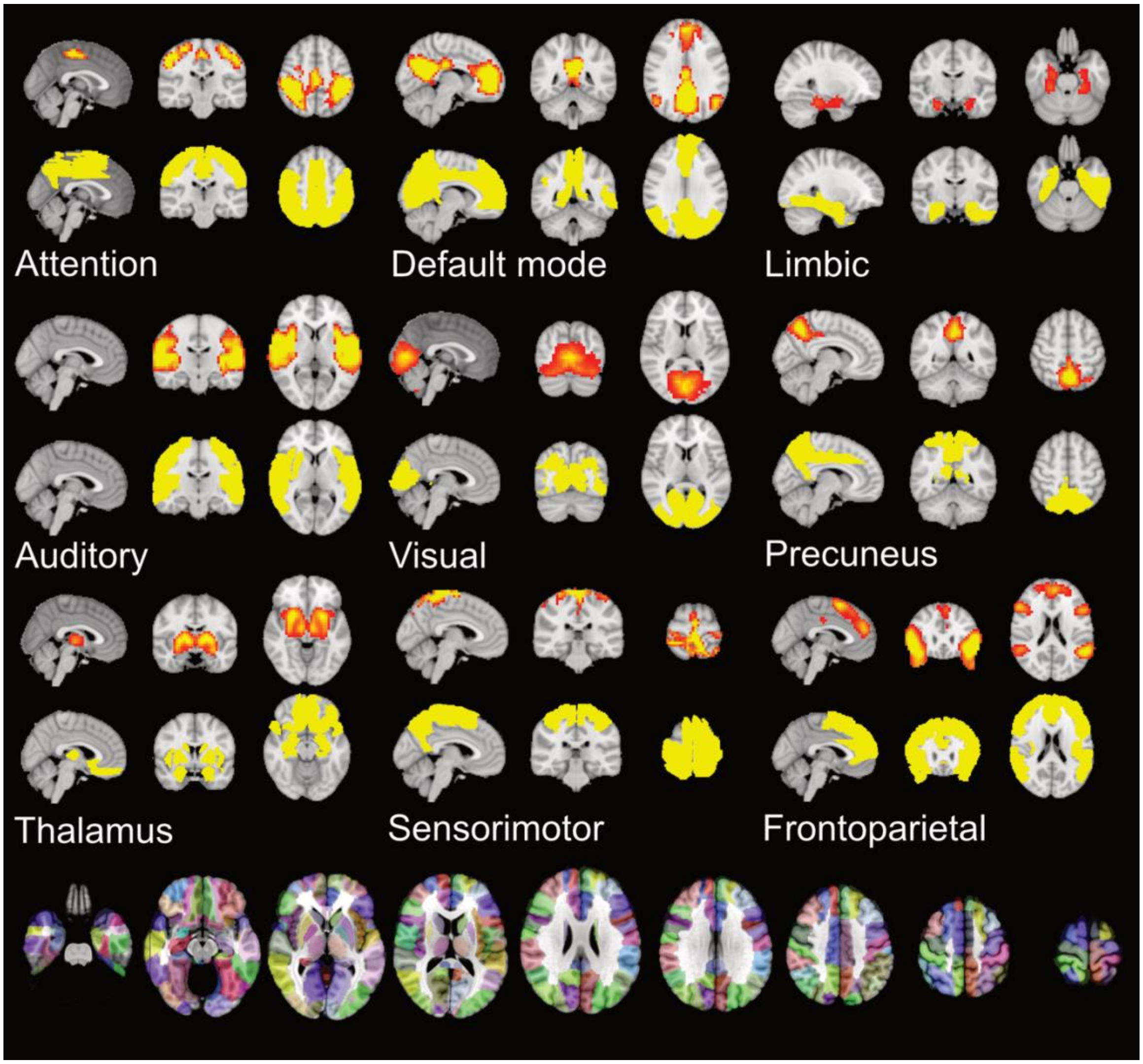

Figure S1. Each network used to restrict the parameter space of the local parameter optimization. Networks are presented in 2 ways: above is the independent component identified from the group ICA on the healthy controls data of the disorders of consciousness dataset. Below is the binary expression

of the independent component in AAL atlas space. At the bottom is the different regions of the AAL atlas.

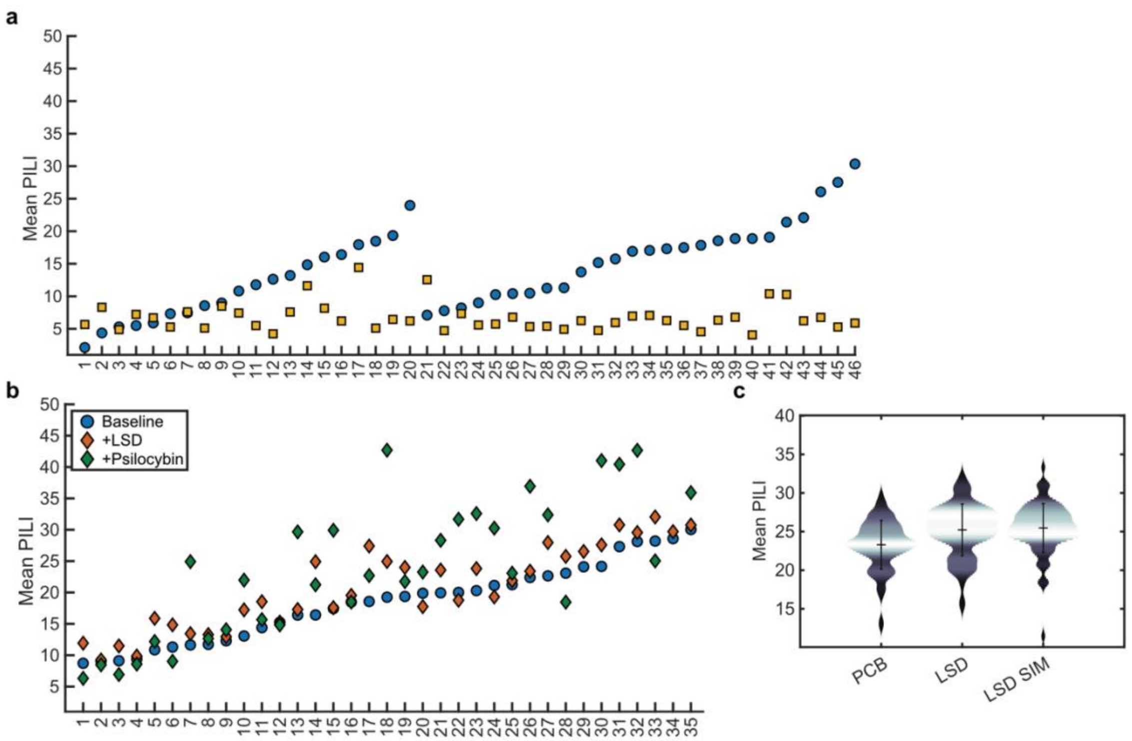

Figure S2. a. Mean PILI values in single patient models individually perturbed by randomly shifting the alpha parameters between -0.3 and +0.3. **b.** Mean PILI values in individual models based on healthy controls. Red diamonds represent LSD and green diamonds represent psilocybin **c.** Mean PILI values from the group level models optimized to Placebo (PCB), LSD and after simulating the administration of LSD on the PCB model.

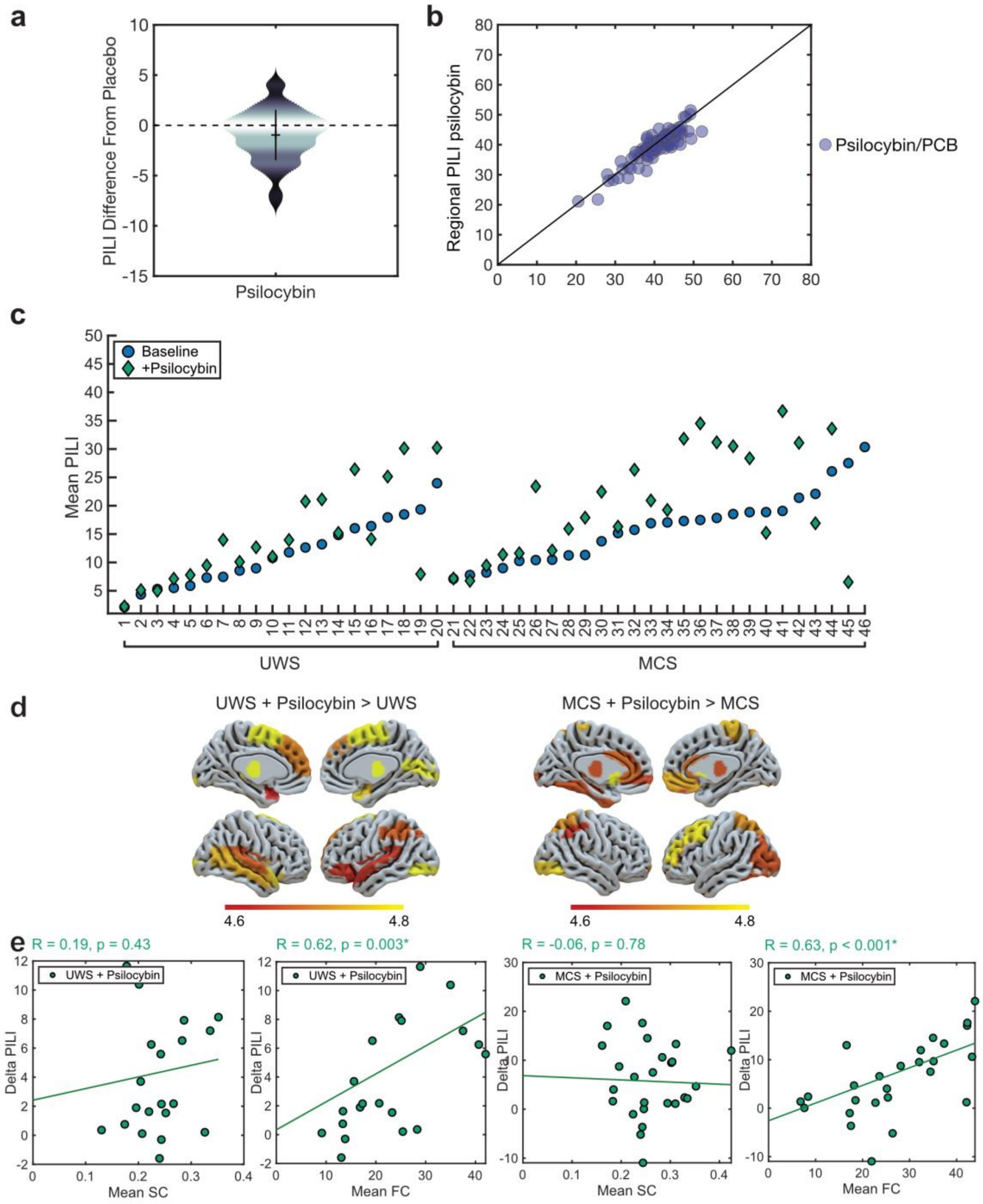

Figure S3. Supplementary analysis using another psilocybin dataset with a between-subject's design. a. Violin plot of the distribution of PILI trials averaged over all brain regions of the group level model of psilocybin. Y axis represents the distance of PILI values in the psilocybin condition compared to the placebo condition. b. Absolute region wise PILI in the psilocybin condition X axis plotted against the placebo condition Y axis. c. Individual patient models before and after simulation of psilocybin. Blue circles are each patient at baseline, green diamonds represent the patient after the simulation of psilocybin. d. Results of region wise t-tests displaying brain regions with significant increases, in PILI

from simulating psilocybin in patients with UWS (left) and MCS patients (right), Bonferroni corrected for multiple comparisons for the 90 brain regions. e. Scatter plots showing correlations between the changing in PILI values as a result of simulating psilocybin (Delta PILI) and the average baseline SC and FC in UWS patients (Left) and MCS patients (Right). Regression lines are indicated in green. Associated correlation coefficients (R) and p-values are stated above, star indicates significance, after Bonferroni correction or the 4 comparisons.

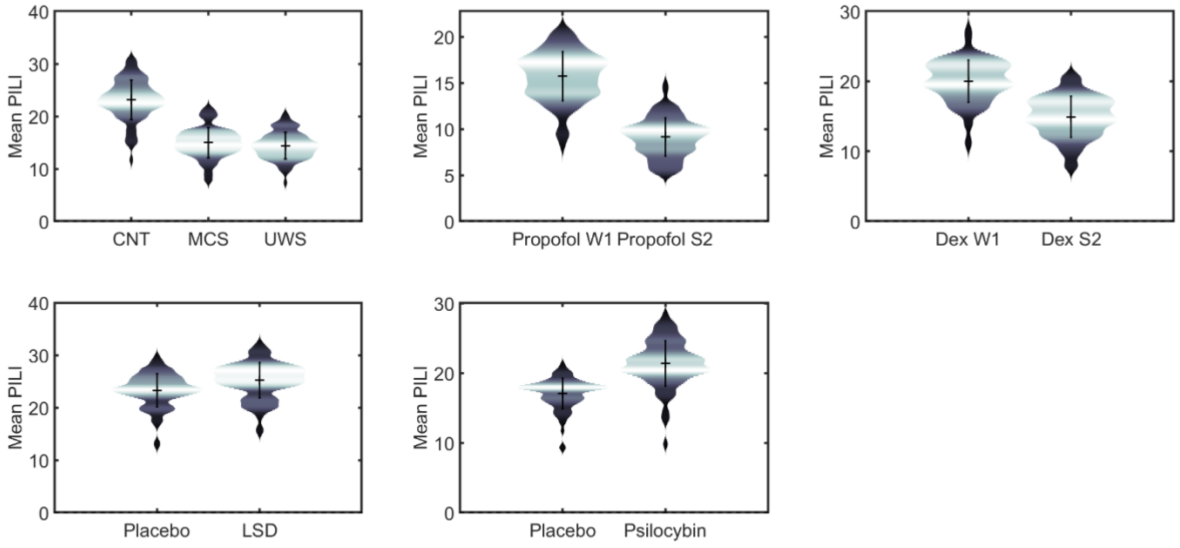

Figure S4.

Violin plots of the distribution of PILI trials averaged over all brain regions of group level models in each state of consciousness. Top. Absolute PILI values in each state of consciousness and the respective comparison condition. W1 refers to normal wakefulness, S2 refers to deep sedation at unresponsive doses.

Subjects	Etiology	TSI (days)	Age	Gender	Best CRS-R total score
UWS 1	NTBI	29	44	M	4
UWS 2	NTBI	50	69	F	5
UWS 3	TBI	8	48	M	2
UWS 4	TBI	283	52	M	6
UWS 5	NTBI	92	74	M	4
UWS 6	NTBI	2890	49	M	6
UWS 7	TBI	24	58	M	4
UWS 8	NTBI	38	50	F	3
UWS 9	NTBI	434	52	M	4
UWS 10	NTBI	129	49	F	4
UWS 11	TBI	3406	31	M	6
UWS 12	NTBI	18	20	M	3
UWS 13	NTBI	304	60	M	6
UWS 14	NTBI	335	40	F	6
UWS 15	NTBI	43	64	M	5
UWS 16	TBI	2013	31	M	4
UWS 17	NTBI	30	44	M	5
UWS 18	NTBI	26	82	M	4
UWS 19	NTBI	40	74	F	7
UWS 20	NTBI	1683	39	F	7
Propofol_2	TBI	533	47	M	4
MCS 1	NTBI	389	59	F	8
MCS 2	Mixed	35	73	M	5
MCS 3	TBI	3034	34	F	12
MCS 4	NTBI	20	52	M	14
MCS 5	TBI	319	73	M	9
MCS 6	TBI	21	25	M	15
MCS 7	TBI	28	65	M	13
MCS 8	NTBI	13	62	M	7
MCS 9	TBI	135	51	M	12
MCS 10	TBI	521	28	M	10
MCS 11	TBI	1294	40	M	11
MCS 12	TBI	25	67	M	11
MCS 13	TBI	641	23	F	10

MCS 14	NTBI	64	29	M	7
MCS 15	NTBI	1482	32	M	14
MCS 16	TBI	407	31	M	9
MCS 17	NTBI	2639	38	M	9
MCS 18	TBI	246	30	M	9
MCS 19	NTBI	242	46	F	7
MCS 20	NTBI	18	74	F	5
MCS 21	TBI	134	66	M	16
MCS 22	TBI	1331	35	M	8
MCS 23	TBI	2690	24	M	14
MCS 24	NTBI	9900	39	M	15
MCS 25	TBI	674	66	F	7
MCS 26	NTBI	396	57	M	8
Propofol_1	NTBI	51	54	M	12

Table S1.

Demographic information of MCS and UWS patients. The table includes condition, etiology (traumatic brain injury (TBI), non-traumatic brain injury (NTBI)), time since injury (TSI), age, gender (F=female, M=male), best Coma Recovery Scale-Revised (CSR-R) total score.

Condition	Global coupling parameter	ATT	AUD	DMN	LIM	FP	PREC	SM	THAL	VIS
CNT	0.77	-0.048	0.095	-0.001	-0.084	-0.043	-0.009	0.111	-0.147	0.025
MCS	0.43	-0.032	0.06	-0.016	0.031	-0.008	0.032	0.072	-0.192	0.007
UWS	0.69	0.011	-0.017	-0.071	-0.052	0.045	-0.039	0.027	-0.185	0.083
Propofol W1	0.69	-0.024	0.048	-0.006	-0.074	-0.061	0.005	0.132	-0.191	0.065
Propofol S2	0.48	-0.022	-0.032	-0.055	-0.03	-0.014	0.062	0.117	-0.187	0.074
Dexmedetomidine W1	0.79	-0.023	0.046	0.014	-0.018	-0.076	0.02	0.113	-0.154	0.054
Dexmedetomidine S2	0.55	-0.002	0.033	0.004	0.03	-0.038	0.02	0.08	-0.126	0.005
LSD placebo	0.93	-0.004	0.051	-0.016	-0.003	-0.064	0.033	0.057	-0.116	0.084
LSD	0.97	-0.003	0.044	0.027	-0.004	-0.067	0.045	0.046	-0.103	0.032
Psilocybin placebo	0.90	-0.005	0.037	0.003	0.026	-0.093	-0.021	0.121	-0.132	0.059
Psilocybin	0.88	-0.051	0.127	0.027	0.004	-0.08	-0.028	0.132	-0.131	-0.034

Table S2. Optimal global coupling and local bifurcation parameters, per network, for the group level models for each dataset. Network abbreviations: ATT – Attention; AUD – Auditory; DMN – Default Mode Network; LIM – Limbic; FP – Frontoparietal; PREC – Precuneus; SM – Sensorimotor; THAL – Thalamus; VIS – Visual.

Subject	Global coupling parameter	ATTENTION	AUD	DMN	LIM	FP	PREC	SM	THAL	VIS
MCS_S01	1.83	-0.144	-0.042	0.138	-0.12	-0.07	-0.066	-0.07	-0.107	-0.005
MCS_S02	0.70	0.092	0.022	-0.046	0.054	-0.043	-0.002	0.007	-0.109	0.065
MCS_S03	0.55	-0.035	0.083	-0.075	0.143	-0.017	0.161	0.009	-0.155	0.039
MCS_S04	0.39	0.064	-0.028	-0.005	0.029	-0.027	0.135	0.039	-0.004	0.055
MCS_S05	0.64	-0.075	0.041	-0.02	0.061	-0.01	0.084	0.122	-0.143	-0.011
MCS_S06	1.21	-0.078	-0.025	-0.039	0.166	0.034	-0.082	0.004	-0.066	-0.022
MCS_S07	0.31	0.164	0.158	0.017	-0.057	0.022	-0.04	-0.074	-0.172	0.003
MCS_S08	1.16	0.011	0.027	-0.047	-0.056	0.024	0.026	0.046	-0.131	-0.129
MCS_S09	1.07	0.076	0.008	-0.098	-0.134	-0.042	-0.039	0.123	-0.17	0.126
MCS_S10	1.22	-0.127	0.033	0.033	-0.054	0.001	-0.039	0.097	-0.184	0.105
MCS_S11	2.00	-0.074	0.132	0.066	-0.033	-0.059	-0.028	0.023	-0.15	-0.009
MCS_S12	0.68	-0.028	0.11	-0.023	-0.095	-0.11	-0.05	0.174	-0.151	0.074
MCS_S13	1.10	-0.049	0.129	-0.014	-0.023	-0.05	0.006	0.107	-0.173	0.027

MCS_S14	1.02	0.016	0.098	-0.076	-0.046	-0.095	-0.023	0.116	-0.143	0.052
MCS_S15	1.41	-0.133	0.073	0.012	-0.117	-0.026	-0.005	0.13	-0.181	0.142
MCS_S16	0.75	0.099	-0.103	-0.042	0.071	0.027	-0.002	-0.033	-0.185	-0.002
MCS_S17	0.36	0.004	-0.032	-0.111	0.018	0.081	-0.105	0.133	-0.07	0.068
MCS_S18	1.43	-0.06	0.099	-0.097	-0.074	-0.055	-0.046	0.121	-0.075	0.085
MCS_S19	1.27	-0.09	-0.083	-0.125	0.073	0.133	0.012	0.05	-0.159	-0.021
MCS_S20	1.60	-0.023	0.069	-0.018	-0.121	-0.025	-0.017	0.125	-0.167	-0.044
MCS_S21	0.71	-0.041	0.117	-0.095	0.187	-0.053	0.104	0.148	-0.153	-0.055
MCS_S22	1.11	0.012	0.103	-0.008	-0.013	-0.128	-0.016	0.167	-0.177	0.023
MCS_S23	1.12	-0.008	0.051	-0.007	-0.021	0.033	-0.031	-0.038	-0.17	-0.021
MCS_S24	2.00	-0.151	-0.006	-0.011	-0.032	0.066	-0.025	0.047	-0.142	0.076
MCS_S25	1.81	-0.021	0.061	-0.064	-0.157	-0.037	-0.035	0.18	-0.178	-0.032
MCS_S26	0.76	0.058	0.149	-0.065	0.058	-0.043	-0.033	0.008	-0.174	0.041
UWS_S01	1.08	-0.051	-0.021	0.008	-0.126	0.029	-0.048	0.033	-0.18	0.097
UWS_S02	0.76	-0.036	-0.027	0.041	-0.027	0.027	0.046	-0.048	-0.105	-0.12
UWS_S03	0.80	0.077	-0.03	-0.012	0.04	-0.004	0.089	-0.06	-0.155	0.026
UWS_S04	0.90	-0.008	-0.035	-0.154	0.032	0.039	-0.012	0.112	-0.159	-0.103
UWS_S05	0.95	0.029	0.061	-0.058	0.045	-0.054	0.016	0.074	-0.149	-0.004
UWS_S06	0.58	-0.011	-0.098	0.008	-0.091	-0.013	-0.061	-0.028	-0.158	-0.128
UWS_S07	0.62	0.02	-0.051	-0.034	0.013	0.064	0.033	-0.092	-0.15	0.047
UWS_S08	2.00	-0.068	-0.024	-0.042	-0.131	0.091	-0.02	0.003	-0.182	0.026
UWS_S09	1.03	-0.157	-0.106	0.049	-0.128	0.069	-0.11	0.038	-0.081	-0.09
UWS_S10	2.00	0.072	-0.09	0.009	0.109	0.061	-0.073	0.002	-0.162	-0.064
UWS_S11	0.81	-0.097	0.024	-0.062	-0.074	0.069	0.016	-0.068	-0.155	0.059
UWS_S12	0.93	-0.001	-0.02	-0.069	-0.127	0.02	-0.081	0.068	-0.071	0.082
UWS_S13	2.00	0.072	0	0.032	-0.04	-0.035	0	-0.071	-0.08	-0.013
UWS_S14	0.81	-0.012	0.08	-0.103	-0.067	-0.031	-0.057	0.157	-0.178	0.012
UWS_S15	0.60	0.11	-0.027	-0.026	-0.08	-0.085	-0.035	0.022	-0.118	0.035
UWS_S16	0.75	-0.001	0.037	-0.013	0.108	0.034	0.026	0.002	-0.163	-0.087
UWS_S17	1.85	0.054	0.092	-0.047	-0.026	-0.093	-0.079	0.136	-0.126	-0.056
UWS_S18	1.66	0.002	0.129	-0.135	-0.08	-0.074	-0.128	0.097	-0.121	0.132
UWS_S19	0.52	0	0.114	-0.064	0.181	-0.039	0.108	0.108	-0.123	-0.043
UWS_S20	1.51	0.022	0.091	0.041	-0.048	-0.151	-0.026	0.128	-0.174	0
CNT_S01	0.92	-0.03	-0.032	0.062	-0.113	0.007	-0.012	-0.069	-0.063	0.017
CNT_S02	1.69	-0.067	0.074	-0.069	-0.06	-0.024	0.009	0.136	-0.145	0.111
CNT_S03	0.65	-0.044	0.071	-0.053	0.024	-0.055	0.084	0.119	-0.15	0.036
CNT_S04	1.03	0.031	-0.025	0.003	-0.041	-0.099	-0.02	0.124	-0.14	0.031
CNT_S05	0.71	-0.05	0.094	-0.023	-0.04	-0.004	0.096	0.056	-0.176	0.014
CNT_S06	0.96	-0.002	-0.019	0.026	0.01	-0.064	0.031	0.065	-0.068	0.019
CNT_S07	1.02	-0.04	0.053	-0.06	-0.158	-0.001	0.014	0.124	-0.185	0.097
CNT_S08	0.93	0.02	0.026	-0.04	-0.099	0.001	0.013	0.039	-0.085	-0.048

CNT_S09	1.22	-0.017	0.081	-0.052	-0.043	-0.044	0.052	0.124	-0.138	-0.063
CNT_S10	1.87	-0.132	0.005	0.041	-0.103	0.012	-0.012	0.042	-0.176	0.103
CNT_S11	1.16	-0.032	0.111	-0.015	-0.066	-0.086	-0.003	0.123	-0.184	0.086
CNT_S12	0.86	0.042	0.022	-0.059	0.006	-0.105	0.048	0.12	-0.178	0.105
CNT_S13	1.18	-0.049	0.139	0.021	-0.037	-0.023	-0.027	0.044	-0.18	-0.053
CNT_S14	0.77	0.011	0.013	-0.026	0.03	-0.096	-0.009	0.149	-0.083	-0.03
CNT_S15	1.46	-0.043	0.115	0.009	-0.131	-0.068	0.052	0.106	-0.191	0.081
CNT_S16	0.96	-0.065	0.073	0.009	-0.058	-0.067	0.059	0.138	-0.058	-0.023
CNT_S17	0.66	-0.064	0.067	-0.046	-0.016	-0.014	-0.093	0.142	-0.11	-0.041
CNT_S18	1.03	-0.02	0.104	-0.067	-0.014	-0.066	-0.027	0.116	-0.141	0.056
CNT_S19	1.59	-0.048	0.088	-0.11	0.007	0.027	-0.075	0.108	-0.171	-0.14
CNT_S20	1.31	-0.109	0.073	-0.019	-0.159	0.02	-0.109	0.138	-0.095	-0.05
CNT_S21	1.23	0.041	0.105	-0.037	0.035	-0.106	0.035	0.066	-0.184	0.07
CNT_S22	1.10	0.033	0.007	-0.055	-0.049	-0.048	-0.087	0.155	-0.185	0.028
CNT_S23	1.27	-0.02	0.034	-0.061	-0.094	-0.006	-0.005	0.1	-0.183	0.104
CNT_S24	0.84	0.032	-0.087	-0.005	-0.157	-0.005	-0.063	0.053	-0.057	0.039
CNT_S25	0.74	-0.038	0.101	-0.063	-0.1	-0.056	-0.075	0.184	-0.184	0.001
CNT_S26	1.12	-0.087	0.027	-0.021	-0.047	-0.05	0.066	0.105	-0.144	0.095
CNT_S27	0.91	-0.056	0.077	-0.05	-0.174	-0.012	-0.018	0.122	-0.159	0.04
CNT_S28	1.58	-0.072	0.094	0.007	-0.045	-0.026	-0.093	0.125	-0.182	0.021
CNT_S29	0.96	-0.074	-0.006	0.07	0.039	-0.06	0.052	0.073	-0.092	0.003
CNT_S30	0.54	-0.019	-0.058	-0.016	-0.091	-0.051	0.05	0.112	-0.052	0.069
CNT_S31	0.88	-0.101	0.02	-0.015	-0.07	0.018	-0.002	0.051	-0.09	0.088
CNT_S32	0.80	-0.014	0.046	-0.061	-0.158	-0.074	-0.035	0.171	-0.174	0.104
CNT_S33	1.39	-0.04	0.075	-0.019	-0.153	-0.045	0.037	0.118	-0.167	0.081
CNT_S34	0.73	-0.02	-0.058	0.003	-0.072	-0.063	-0.042	0.165	-0.107	0.047
CNT_S35	0.88	0.032	0.019	-0.046	-0.124	-0.081	-0.116	0.156	-0.137	0.008

Table S3. Optimal global coupling and local bifurcation parameters, per network, for the individual level patient models for each dataset. Network abbreviations: ATT – Attention; AUD – Auditory; DMN – Default Mode Network; LIM – Limbic; FP – Frontoparietal; PREC – Precuneus; SM – Sensorimotor; THAL – Thalamus; VIS – Visual.

Condition	UWS – CNT	MCS – CNT	Propofol – wakefulness	Dexmedetomidine – wakefulness	LSD – placebo	Psilocybin – Placebo
Whole brain Average	-0.52	-0.48	-0.54	-0.32	0.11	0.28
Attention	-2.37	-2.07	-2.26	-1.29	0.41	1.36
Auditory	-2.25	-2.02	-2.37	-1.36	0.41	1.14
Default Mode	-2.62	-2.46	-2.41	-1.54	0.62	1.55
Limbic	-1.48	-1.56	-1.78	-1.17	0.38	0.23
Frontoparietal	-3.5	-2.57	-3.23	-2.03	0.78	2.01
Precuneus	-1.25	-1.2	-1.18	-0.69	0.4	0.57
Sensorimotor	-1.89	-1.65	-1.77	-1.08	0.42	1.11
Thalamus	-2.84	-2.44	-2.81	-1.81	0.43	1.18
Visual	-2.04	-2.14	-1.93	-1.32	0.29	0.74

Table S4.

Whole brain average and network-wise differences in PILI values from the group level models to the respective comparison condition, assessed by Cohen's d effect size.

Drugs	Global coupling parameter	ATT	AUD	DMN	LIM	FP	PREC	SM	THAL	VIS
LSD	0.04	0.0006	-0.0065	0.043	-0.0016	-0.0024	0.0121	-0.0107	0.0125	-0.0518
Psilocybin	-0.02	-0.0490	0.0903	0.0237	-0.0228	0.0132	-0.0074	0.0103	0.0008	-0.0942

Table S5.

Parameter changes representing the shift in parameters that represent the simulation of LSD and psilocybin. Network abbreviations: ATT – Attention; AUD – Auditory; DMN – Default Mode Network; LIM – Limbic; FP – Frontoparietal; PREC – Precuneus; SM – Sensorimotor; THAL – Thalamus; VIS – Visual.

Group		Brain networks								
		Attention	Auditory	Default mode	Limbic	Frontoparietal	Precuneus	Sensorimotor	Thalamus	Visual
UWS+ LSD	Z-statistic	2.77	2.69	2.61	2.23	2.69	2.46	**2.95**	2.73	2.73
	P-value	0.005	0.007	0.008	0.028	0.007	0.014	**0.003**	0.006	0.006
UWS+ Psilocybin	Z-statistic	**3.92**	**3.92**	**3.85**	**3.62**	**3.88**	**3.32**	**3.92**	**3.88**	**3.88**
	P-value	**<0.001**	**<0.001**	**<0.001**	**<0.001**	**<0.001**	**<0.001**	**<0.001**	**<0.001**	**<0.001**
MCS + LSD	Z-statistic	**4.153**	**4.026**	**4.229**	**3.518**	**4.127**	**4.127**	**4.076**	**4.026**	**3.899**
	P-value	**<0.001**	**<0.001**	**<0.001**	**<0.001**	**<0.001**	**<0.001**	**<0.001**	**<0.001**	**<0.001**
MCS + psilocybin	Z-statistic	**3.797**	**3.584**	**3.847**	**3.70**	**3.746**	**3.797**	**3.721**	**3.797**	**3.721**
	P-value	**<0.001**	**<0.001**	**<0.001**	**<0.001**	**<0.001**	**<0.001**	**<0.001**	**<0.001**	**<0.001**

Table S6.

Network wise changes in PILI in each group and condition from the individualized models. Wilcoxon signed-rank tests. Bold indicates significance after Bonferroni correction for the 9 networks.

Network	F statistic	ANOVA p-Value	Post-hoc Comparison	Mean Difference	p-Value (Post-hoc)
Mean FC	4.493	0.0142	MCS vs. UWS	-3.491	0.419
			MCS vs. CNT	-11.106	0.202
			UWS vs. CNT	-16.560	0.012
Attention	5.071	0.0433	MCS vs. UWS	0.0567	0.4538
			MCS vs. CNT	-0.0554	0.3711
			UWS vs. CNT	-0.1122	0.0356
Auditory	13.232	<0.001	MCS vs. UWS	0.0847	0.1674
			MCS vs. CNT	-0.1086	0.0234
			UWS vs. CNT	-0.1934	0.0001
Default Mode	7.177	0.0082	MCS vs. UWS	0.0934	0.0658
			MCS vs. CNT	-0.0291	0.6952
			UWS vs. CNT	-0.1225	0.0062
Limbic	6.343	0.0088	MCS vs. UWS	0.0207	0.8637
			MCS vs. CNT	-0.0856	0.0427
			UWS vs. CNT	-0.1062	0.0168
Frontoparietal	6.039	0.0139	MCS vs. UWS	0.0658	0.2917
			MCS vs. CNT	-0.0569	0.2974
			UWS vs. CNT	-0.1227	0.0104
Precuneus	10.076	0.0014	MCS vs. UWS	0.0558	0.3229
			MCS vs. CNT	-0.0774	0.0612
			UWS vs. CNT	-0.1333	0.0013
Sensorimotor	6.899	0.0123	MCS vs. UWS	0.0585	0.4098
			MCS vs. CNT	-0.07	0.1899
			UWS vs. CNT	-0.1285	0.0105
Thalamus	10.496	<0.001	MCS vs. UWS	0.0652	0.2111
			MCS vs. CNT	-0.0823	0.0414
			UWS vs. CNT	-0.1474	0.0003
Visual	13.232	<0.001	MCS vs. UWS	0.0541	0.288
			MCS vs. CNT	-0.1211	0.0006
			UWS vs. CNT	-0.1752	<0.001

Table S7.

Results of one-way ANOVA of functional connectivity in 9 resting state networks and the overall average FC in overall brain regions between MCS and UWS patients and healthy controls.

Metric	F-statistic	ANOVA p-Value	Post-hoc Comparison	Mean Difference	p-Value (Post-hoc)
Mean SC	1.294	0.1524	MCS vs. UWS	0.0225	0.4114
			MCS vs. CNT	-0.0106	0.7833
			UWS vs. CNT	-0.0331	0.1303
Graph strength	6.305	0.0028	MCS vs. UWS	0.0339	0.1207
			MCS vs. CNT	-0.0242	0.2596
			UWS vs. CNT	-0.0581	0.0018
Global efficiency	4.819	0.0104	MCS vs. UWS	0.0014	0.1994
			MCS vs. CNT	-0.001	0.3497
			UWS vs. CNT	-0.0023	0.0073
Local efficiency	2.390	0.0978	MCS vs. UWS	-0.0001	0.6021
			MCS vs. CNT	-0.0002	0.0800
			UWS vs. CNT	-0.0001	0.5538
Centrality	5.105	0.0081	MCS vs. UWS	-9.916	0.069
			MCS vs. CNT	3.4527	0.6602
			UWS vs. CNT	13.3687	0.0063
Characteristic Path length	39.149	<0.001	MCS vs. UWS	-1.4957	<0.001
			MCS vs. CNT	1.2375	<0.001
			UWS vs. CNT	2.7332	<0.001
Fractional anisotropy	67.491	<0.001	MCS vs. UWS	0.012	0.1204
			MCS vs. CNT	-0.0486	<0.001
			UWS vs. CNT	-0.0606	<0.001

Table S8.

Results of one-way ANOVA of structural connectivity graph metrics and the overall average structural connectivity over all brain regions between MCS and UWS patients and healthy controls.

Group	Condition	Centrality	Characteristic path length	Fractional anisotropy	Global efficiency	Local efficiency	Graph strength
UWS + LSD	R-value	-0.524	-0.384	0.567	0.541	-0.088	0.534
	P-value	0.018	0.095	0.009	0.014	0.711	0.015
UWS + psilocybin	R-value	-0.328	-0.382	0.388	0.371	0.494	0.493
	P-value	0.158	0.096	0.091	0.107	0.027	0.027
MCS + LSD	R-value	-0.260	-0.124	-0.150	0.688	-0.033	0.095
	P-value	0.200	0.547	0.462	0.139	0.872	0.645
MCS + psilocybin	R-value	-0.396	0.309	-0.186	-0.111	-0.140	-0.098
	P-value	0.045	0.124	0.363	0.591	0.494	0.633

Table S9.

Results of correlational analyses between baseline structural graph theory metrics and the changes in PILI values as a result of simulating LSD and psilocybin in patients with UWS and patients in the MCS.

Group	Condition	Attention	Auditory	Default Mode	Limbic	Frontoparietal	Precuneus	Sensorimotor	Thalamus	Visual
UWS + LSD	R-value	0.035	0.184	0.225	0.199	0.340	0.159	0.050	0.353	0.395
	P-value	0.885	0.438	0.334	0.401	0.142	0.502	0.834	0.127	0.085
UWS + psilocybin	R-value	0.203	0.348	0.365	0.371	0.465	0.152	0.140	0.328	0.451
	P-value	0.391	0.132	0.114	0.107	0.034	0.522	0.558	0.158	0.046
MCS + LSD	R-value	**0.622***	0.466	**0.567***	0.298	**0.569***	0.521	**0.592***	0.531	0.266
	P-value	**0.0007***	0.017	**0.003***	0.139	**0.002***	0.006	**0.001***	0.005	0.189
MCS + psilocybin	R-value	0.467	0.435	**0.598***	0.347	0.534	0.468	0.427	0.520	0.302
	P-value	0.016	0.027	**0.001***	0.082	0.005	0.016	0.028	0.007	0.134

Table S10.

Correlational analyses between the strength of FC in each resting state network at baseline and the change in PILI as a result of simulating psilocybin and LSD in patients with UWS and MCS. Star represents significance after Bonferroni correction for the 9 networks.

Supplementary Materials References

[1] J. T. Giacino, K. Kalmar, J. Whyte, *Arch. Phys. Med. Rehabil.* **2004**, *85*, 2020.

[2] P. Boveroux, A. Vanhaudenhuyse, M.-A. Bruno, Q. Noirhomme, S. Lauwick, A. Luxen, C. Degueldre, A. Plenevaux, C. Schnakers, C. Phillips, J.-F. Brichant, V. Bonhomme, P. Maquet, M. D. Greicius, S. Laureys, M. Boly, *Anesthesiology* **2010**, *113*, 1038.

[3] P. Guldenmund, A. Vanhaudenhuyse, R. D. Sanders, J. Sleigh, M. A. Bruno, A. Demertzi, M. A. Bahri, O. Jaquet, J. Sanfilippo, K. Baquero, M. Boly, J. F. Brichant, S. Laureys, V. Bonhomme, *Br. J. Anaesth.* **2017**, *119*, 674.

[4] M. A. E. Ramsay, T. M. Savege, B. R. J. Simpson, R. Goodwin, *Br. Med. J.* **1974**, *2*, 656.

[5] R. L. Carhart-Harris, S. Muthukumaraswamy, L. Roseman, M. Kaelen, W. Droog, K. Murphy, E. Tagliazucchi, E. E. Schenberg, T. Nest, C. Orban, R. Leech, L. T. Williams, T. M. Williams, M. Bolstridge, B. Sessa, J. McGonigle, M. I. Sereno, D. Nichols, P. J. Hellyer, P. Hobden, J. Evans, K. D. Singh, R. G. Wise, H. V. Curran, A. Feilding, D. J. Nutt, *Proc. Natl. Acad. Sci. U. S. A.* **2016**, *113*, 4853.

[6] R. L. Carhart-Harris, D. Erritzoe, T. Williams, J. M. Stone, L. J. Reed, A. Colasanti, R. J. Tyacke, R. Leech, A. L. Malizia, K. Murphy, P. Hobden, J. Evans, A. Feilding, R. G. Wise, D. J. Nutt, *Proc. Natl. Acad. Sci.* **2012**, *109*, 2138.

[7] N. L. Mason, K. P. C. Kuypers, F. Müller, J. Reckweg, D. H. Y. Tse, S. W. Toennes, N. R. P. W. Hutten, J. F. A. Jansen, P. Stiers, A. Feilding, J. G. Ramaekers, *Neuropsychopharmacology* **2020**, *45*, 2003.
